# Abnormalities in cobalamin status in dogs with congenital portosystemic shunt before and after shunt closure

**DOI:** 10.1093/jvimsj/aalag277

**Published:** 2026-11-13

**Authors:** Anna-Lena Proksch, Tim Kunath, Romy Monika Heilmann, Matthias Alois Schneider, Maximilian Kaline, Vanessa Dreller, Judith Langenstein, Natali Bauer, Andreas Moritz

**Affiliations:** Clinic for Small Animals, Internal Medicine, JLU Giessen, 35392 Giessen, Germany; Department for Small Animals, College of Veterinary Medicine, University of Leipzig, 04103 Leipzig, Germany; Department for Small Animals, College of Veterinary Medicine, University of Leipzig, 04103 Leipzig, Germany; Clinic for Small Animals, Internal Medicine, JLU Giessen, 35392 Giessen, Germany; Clinic for Small Animals, Internal Medicine, JLU Giessen, 35392 Giessen, Germany; Clinic for Small Animals, Internal Medicine, JLU Giessen, 35392 Giessen, Germany; Antech Lab Germany GmbH, 68519 Viernheim, Germany; Clinic for Small Animals, Internal Medicine, JLU Giessen, 35392 Giessen, Germany; Clinic for Small Animals, Internal Medicine, JLU Giessen, 35392 Giessen, Germany

**Keywords:** canine, folate, vitamin B12, homocysteine, MMA/methylmalonic acid, transcobalamin

## Abstract

**Background:**

Cobalamin dysvitaminosis occurs with congenital portosystemic shunt (cPSS). Limited data exist on the effects of cPSS-type, cPSS-closure, and association with hyperammonemia.

**Hypothesis/Objectives:**

Cobalamin and folate status differ between healthy and cPSS-dogs, before and after cPSS-closure, between complete and incomplete cPSS-closure, and cPSS-types. Cobalamin dysvitaminosis is associated with hyperammonemia, which ameliorates after cPSS-closure.

**Animals:**

Eighty-three dogs.

**Methods:**

Concentrations of cobalamin, transcobalamin (TCB), metabolites (homocysteine [HCy], serum [sMMA], urinary [uMMA:crea] methylmalonic acid), folate, and blood ammonia were prospectively compared between 44 cPSS-dogs and 39 age and body weight matched controls. Compared were 32 out of 44 intrahepatic (cIPSS) and 12 out of 44 extrahepatic (cEPSS) cPSS-dogs, before (PSS-0) and 12 weeks (PSS-12) after cPSS-closure, and complete versus incomplete cPSS-closure.

**Results:**

Hypocobalaminemia occurred in 2 out of 44 PSS-0-dogs (cIPSS) and hypercobalaminemia in 8 out of 44 PSS-0-dogs (5/12 cEPSS, 3/32 cIPSS). Of 34 normocobalaminemic PSS-0-dogs, in 47% available HCy samples concentrations were below the reference interval, whereas sMMA and uMMA:crea were higher in 61% and 27%. PSS-0-dogs had lower HCy, higher sMMA and uMMA:crea, and lower folate concentrations than controls (each *P* < .0001). Hyperammonemia occurred in 35 out of 43 PSS-0-dogs, but ammonia did not correlate with any variable. Of 32 out of 38 normocobalaminemic PSS-12-dogs, 31% had supraphysiologic sMMA and 10% supraphysiologic uMMA:crea concentrations. PSS-12-dogs had lower cobalamin (*P* = .0001) and TCB (*P* = .014), higher HCy (*P* < .0001), lower uMMA:crea (*P* = .0002), and higher folate concentrations (*P* < .0001) than PSS-0-dogs. At PSS12, cIPSS-dogs had significantly higher sMMA concentrations (*P* = .0001) than cEPSS-dogs. Lower sMMA (*P* = .0001) and uMMA:crea (*P* = .003) were more common with complete than incomplete cPSS-closure.

**Conclusions and clinical importance:**

Dysvitaminosis is common in cPSS-dogs persisting in some cPSS-dogs after cPSS-closure.

**Abstract (German):**

**Hintergrund**

Cobalamin-Dysvitaminosen können bei Hunden mit angeborenem portosystemischem Shunt (cPSS) auftreten. Daten über den Einfluss von Shunttyp und -verschluss auf den Vitaminstatus sowie dessen Zusammenhang mit einer Hyperammonämie sind begrenzt.

**Hypothese/Ziele**

Cobalamin- und Folsäurestatus unterscheiden sich zwischen gesunden und cPSS-Hunden, vor und nach Shuntverschluss, zwischen vollständigem und unvollständigem Verschluss sowie zwischen Shunttypen. Cobalamin-Dysvitaminosen sind mit Hyperammonämie assoziiert und bessern sich nach Shuntverschluss.

**Methoden**

Cobalamin, Transcobalamin, Vitamin-abhängige Metaboliten (Homocystein, Serum-Methylmalonsäure [sMMA], Urin-Methylmalonsäure/Kreatinin-Quotient [uMMA:crea]), Folsäure und Blutammoniak wurden prospektiv zwischen 44 cPSS-Hunden und 39 alters- und körpergewichtsangepassten Kontrollhunden verglichen. Verglichen wurden Hunde mit intrahepatischen (cIPSS; 32/44) und extrahepatischen cPSS (cEPSS; 12/44), vor (PSS-0) und zwölf Wochen nach Shuntverschluss (PSS-12) sowie vollständiger und unvollständiger Shuntverschluss.

**Ergebnisse**

Hypocobalaminämie bestand bei 2/44 PSS-0-Hunden (beide cIPSS), Hypercobalaminämie bei 8/44 (5/12 cEPSS, 3/32 cIPSS). Bei 34 normocobalaminämischen PSS-0-Hunden lagen 47% der verfügbaren Homocysteinwerte unterhalb des Referenzintervalls; sMMA- und uMMA-Konzentrationen waren bei 61% beziehungsweise 27% erhöht. PSS-0-Hunde hatten niedrigere Homocystein- und Folsäurekonzentrationen sowie höhere sMMA- und uMMA-Konzentrationen als Kontrollhunde (jeweils *P* < 0,0001). Hyperammonämie bestand bei 35/43 PSS-0-Hunden, Ammoniak korrelierte jedoch mit keiner untersuchten Variable. Unter den 32/38 normocobalaminämischen PSS-12-Hunden waren die sMMA- und uMMA:crea-Konzentrtaionen bei 31% und 10% erhöht. Nach Verschluss waren die Konzentrationen von Cobalamin (*P* = 0,0001), Transcobalamin (*P* = 0,014) und uMMA:crea (*P* = 0,0002) niedriger, von Homocystein und Folsäure höher gegenüber PSS-0 (jeweils *P* < 0,0001). cIPSS-Hunde hatten höhere sMMA-Konzentrationen als cEPSS-Hunde (*P* = 0,0001). Niedrigere sMMA- (*P* = 0,0001) und uMMA:crea-Konzentrationen (*P* = 0,003) waren bei vollständigem Verschluss häufiger als bei Teilverschluss.

**Schlussfolgerung und klinische bedeutung**

Dysvitaminosen sind bei cPSS-Hunden häufig und persistieren bei einigen Hunden nach Shuntverschluss.

## Introduction

Cobalamin and folate are essential cofactors for the enzyme methionine synthase, whereas only cobalamin is required for methylmalonyl-CoA mutase activity.^1^ Both vitamins are critical for hematopoiesis, synthesis of nucleic acids and proteins, and nerve sheath myelination, thus, promoting growth.^2–4^ Clinical consequences of deficiency can be detrimental, causing failure to thrive, psychomotor developmental delay, seizures, and potentially death.^5–8^ Cobalamin deficiency can cause malfunction of both cobalamin-dependent enzymes with a concurrent increase in homocysteine (HCy) and methylmalonic acid (MMA), while folate deficiency increases HCy concentrations only.^1^^,^^2^^,^^9^ Supraphysiologic HCy and MMA concentrations can be associated with direct neurotoxicity: high MMA concentrations can contribute to hyperammonemia as methylmalonyl-CoA indirectly inhibits the carbamoyl-phosphate synthetase, which promotes ammonia entry into the urea cycle.^10^^,^^11^ Because of hepatic hypoperfusion and dysfunction, dogs with congenital portosystemic shunt (cPSS) are predisposed to hyperammonemia-related hepatic encephalopathy.^12^^,^^13^ Furthermore, the majority of a small group of dogs with extrahepatic cPSS (cEPSS) had alterations of cobalamin metabolism, namely hypercobalaminemia and variable serum MMA (sMMA) concentrations, persisting 3 months after cPSS-closure.^14^ Hypercobalaminemia remains a neglected dysvitaminosis that in people and dogs might be associated with gastrointestinal and hepatic disease.^15^^,^^16^ Yet, total cobalamin (tCbl) concentrations do not invariably reflect cobalamin availability at the cellular level because serum tCbl concentrations mostly reflect the biologically inactive form (cobalamin bound to haptocorrin). In some people with hypercobalaminemia, paradoxical clinical signs of deficiency are described.^15^^,^^17^^,^^18^ In people, routine assessment of the cobalamin status includes measurement of active cobalamin: holo-transcobalamin (cobalamin bound to transcobalamin [TCB]).^19^ In dogs, measurement of holo-TCB is not available and little is reported about TCB concentrations.^20^ Data on cobalamin and its markers of metabolism (markersB_12_met: HCy, MMA) are scarce in dogs with liver disease, particularly with cPSS.^14^^,^^16^^,^^21^^,^^22^ Furthermore, there is no published data on whether possible derangements in the cobalamin status in cPSS-dogs are associated with hyperammonemia and whether cobalamin status differs between dogs with cEPSS and intrahepatic cPSS (cIPSS).

Aims of this study were: (1) compare cobalamin status (tCbl, TCB, markersB_12_met) and folate concentrations between PSS-0 and healthy dogs; (2) assess changes between PSS-0-dogs and PSS-12-dogs; (3) evaluate differences between cIPSS-dogs and cEPSS-dogs before and after cPSS-closure; (4) assess differences between cPSS-12-dogs with complete and incomplete cPSS-closure (overall and by cPSS-type); and (5) evaluate associations between ammonia and cobalamin status.

## Materials and methods

This prospective study included privately owned dogs after informed owners’ consent. The local ethics committee provided ethical approval.

Diagnosis of cPSS included the combination of hyperammonemia (point-of-care blood ammonia device [PocketChem BA PA-4140, Arkray Europe, B.V., Amstelveen, The Netherlands]), abnormal serum bile acid (SBA) stimulation test and presence of cPSS confirmed by abdominal ultrasonography or computed tomography. Medical stabilization before cPSS-closure (first study point, PSS-0) involved a minimum of 4 weeks of appropriate diet, oral lactulose, and proton pump inhibitors. Medical treatment was continued until recheck approximately 12 weeks after cPSS-closure (second study point, PSS-12). A standard questionnaire regarding the dog’s general health and specific questions related to cPSS were answered, and basic laboratory data (hematology, biochemistry, C-reactive protein, urinalysis) were required. Exclusion criteria were declined cPSS-closure, concurrent diseases, strict feeding a vegan diet, vitamin and antimicrobial treatment within 3 months before cPSS-closure and until re-check. At PSS-12, a repeated study questionnaire, clinical examination, basic laboratory data, SBA stimulation test, blood ammonia, and abdominal ultrasonography (color Doppler flow measurements) assessing residual shunt flow were required.

Healthy age and body weight matched control dogs were assessed by a standard questionnaire.^23^ Abnormalities in physical examination, basic laboratory data or suspicion of an underlying disease (questionnaire) or receiving a vegan diet, vitamin supplementation or systemic antimicrobial treatment within the last 3 (puppies) or 6 months (adult dogs) led to exclusion.

Serum tCbl and folate concentrations were analyzed by a chemiluminescent enzyme immunoassay (IMMULITE^®^ 2000).^23–27^ Serum TCB concentrations were measured by a validated in-house direct sandwich enzyme-linked immunosorbent assay.^20^ Method (liquid chromatography/tandem mass spectrometry (LC–MS/MS)) and reference intervals (RI) to assess markersB_12_met (HCy, sMMA, uMMA:crea) were previously described.^23^ For adult dogs, the tCbl RI used was 178.5-805.1 pmol/L (normocobalaminemia).^23^ Hypo- and hypercobalaminemia were defined as below or above the RI, respectively. For cPSS-puppies, an in-house puppy-specific tCbl RI was applied.

Statistical analyses were performed using GraphPad Prism v5. To compare groups, a Mann–Whitney-*U* test, unpaired or paired *t-*test was applied based on normality distribution. Group comparisons comprised tCbl, TCB, markersB_12_met, and folate concentrations between healthy dogs and cPSS-dogs, cPSS-dogs before and after cPSS-closure (each including comparisons between cIPSS-dogs and cEPSS-dogs), and PSS-12-dogs with and without complete cPSS-closure. At PSS-0, possible correlations between tCbl and TCB, between ammonia and tCbl, and between ammonia and TCB, HCy, sMMA, or uMMA:crea, and between HCy and folate, albumin, or creatinine concentrations were assessed by Spearman’s correlation. Significance was set as *P* < .05.

## Results

The study included 44 cPSS-dogs presented for a cPSS-closure procedure of which 38/44 (86%) dogs were re-presented at PSS-12. In 32/44 (73%) dogs, a cIPSS and in 12/44 (27%), a cEPSS was diagnosed. At PSS-12, 29/32 (91%) cIPSS-dogs and 9/12 (75%) cEPSS-dogs were re-examined. Matched by age and body weight, 39 healthy dogs were included as controls. Median age in both groups was 1 year (*P* = .502; range 0.4-7.0 (healthy dogs) and 0.4-7.8 years (cPSS-dogs)), including 16 (41%) and 19 (43%) puppies (aged < 1 year), respectively. Median body weight in cPSS-dogs was 17.7 kg (range: 3.9-40 kg) and 17.3 kg (range: 3.8-46.5 kg) in controls (*P* = .796). Golden (*n* = 7) and Labrador retrievers (*n* = 6) were the most common breeds in the cPSS group, whereas mixed breed dogs (*n* = 14) were most common among healthy controls (Table 1). In some dogs, sample material was insufficient for the analysis of all variables. Data on available samples in healthy dogs and cPSS-dogs at PSS-0 and PSS-12, and cPSS-dogs with complete and incomplete cPSS-closure are listed in Table 2.

**Table 1:** Signalment of healthy dogs and cPSS-dogs before cPSS-closure.

	**Healthy dogs (*n* = 39)**	**cPSS-dogs (*n* = 44)**
		cEPSS: *n* = 12 (27%) cIPSS: *n* = 32 (73%)
**Age**	Median: 1 year (range: .5-7.0 years)	Median: 1 year (range: .4-7.8 years)
**Puppies (<1 year)**	*n* = 16 (41%)	*n* = 19 (43%)
**Sex and neuter status**	Puppies: Males: 6 IM Females: 10 IF Adult dogs: Males: 7 IM, 5 NM Females: 8 IF, 3 SF	Puppies: Males: 7 IM, 1 NM Females: 11 IF Adult dogs: Males: 18 IM, 1 NM, Females: 5 IM, 1 SF
**Body weight**	Median: 17.3 kg (range: 3.8- 46.5 kg)	Median: 17.7 kg (range: 3.9- 40 kg)
**Breed**	Australian shepherd: 4 Bernese mountain dog: 1 Border collie: 1 Border terrier: 1 Boxer: 1 Dachshund: 2 Flat coated retriever: 1 French bulldog: 1 Golden retriever: 4 Irish terrier: 1 Labrador retriever: 4 Maltese: 1 Mixed breed: 14 Mudi: 1 Pug: 1 Siberian husky: 1	Australian shepherd: 3 Bernese mountain dog: 4 Bichon frise: 1 Bolonka zwetna: 1 Dachshund: 3 Golden retriever: 7 Havanese: 1 Hovawart: 1 Hungarian viszla: 1 Irish wolfhound: 1 Jack Russel terrier: 1 Labrador retriever: 6 Miniature schnauzer: 1 Mixed breed: 9 Old English sheepdog: 1 Pug: 1 Siberian husky: 1 Yorkshire terrier: 1

Abbreviations: cEPSS = congenital extrahepatic portosystemic shunt; cIPSS = congenital intrahepatic portosystemic shunt; cPSS = congenital portosystemic shunt; IF = intact female; IM = intact male, NM = neutered male; SF = spayed female

**Table 2:** Available data for cobalamin status variables and folate measurements in healthy dogs and cPSS-dogs.

	**Healthy dogs** ***n* = 39**	**PSS-0-dogs** ***n* = 44**	**PSS-12-dogs** ***n* = 38**	**PSS-12-dogs with complete closure** ***n* = 14**	**PSS-12-dogs with incomplete closure** ***n* = 24**
**tCbl**	39	44	38	14	24
**TCB**	36 21/36 below detection limit	39 18/39 below detection limit	35	12	23
**HCy**	39	44	38	14	24
**sMMA**	34	38	35	13	22
**uMMA:crea**	39	43	37	14	23
**folate**	24	39	35	12	23

Abbreviations: HCy = homocysteine, PSS-0 = dogs with portosystemic shunt at the time of presentation for cPSS-closure; PSS-12 = dogs with portosystemic shunt presented approximately 12 weeks after cPSS-closure; sMMA = methylmalonic acid in serum, tCbl = total cobalamin, TCB = transcobalamin, uMMA:crea = urinary methylmalonic acid-to-urinary-creatinine-ratio.

Cobalamin analyte and folate concentrations in cPSS-0-dogs and healthy dogs.

Cobalamin status derangements were common in cPSS-dogs (Table 3). Hypercobalaminemia occurred in 8 out of 44 (18%) PSS-0-dogs, and 3 out of 8 (38%) and 1 out of 8 (13%) of those had supraphysiologic sMMA and uMMA:crea concentrations, respectively. Transcobalamin concentrations were below the detection limit (<80 aU/L) in 6/8 (75%) hypercobalaminemic PSS-0-dogs. In PSS-0-dogs, there was neither a correlation between tCbl and TCB nor between HCy and folate or albumin (Table 4) or between ammonia and TCbl, TCB, or markersB_12_met. There was, however, a moderate correlation between HCy and blood creatinine.

**Table 3:** Prevalence of cobalamin status derangements in cPSS-dogs before (PSS-0) and 12 weeks after cPSS-closure (PSS-12).

	**Hypocobalaminemia**		**Normocobalaminemia**		**Hypercobalaminemia**	
	PSS-0	PSS-12	PSS-0	PSS-12	PSS-0	PSS-12
**Number of dogs**	2/44 (5%)	3/38 (8%)	34/44 (77%)	32/38 (84%)	8/44 (18%)	3/38 (8%)
Number of abnormal parameters out of available samples according to the respective total cobalamin concentration						
**TCB ↓**	1/2	0/3	11/29 (38%)	19/31 (61%)	6/8 (75%)	1/2
**TCB ↑**	0/2	0/3	10/29 (34%)	6/31 (19%)	1/8 (13%)	0/2
**HCy ↓**	0/2	0/3	16/34 (47%)	3/32 (9%)	5/8 (63%)	0/3
**HCy ↑**	0/2	0/3	1/34 (3%)	1/32 (3%)	0/8	0/3
**sMMA ↓**	0/2	0/3	0/28	1/29 (3%)	0/8	0/3
**sMMA ↑**	1/2	3/3	17/28 (61%)	9/29 (31%)	3/8 (38%)	1/3
**uMMA:crea ↑**	1/2	1/3	9/33 (27%)	3/31 (10%)	1/8 (13%)	0/3

Note: Sample specimen were not available in all normocobalaminemic and hypercobalaminemic dogs. Numbers refer to abnormal parameters out of available sample specimen from a total of 44 and 38 cPSS-dogs at PSS-0 and PSS-12, respectively.

↓ concentration below reference interval, ↑ concentration above reference interval, cPSS: congenital portosystemic shunt, HCy: total homocysteine, PSS-0: before cPSS-closure, PSS-12: 12 weeks after cPSS-closure, sMMA: serum methylmalonic acid, tCbl: total cobalamin, TCB: transcobalamin, uMMA:crea: urinary methylmalonic acid-to-creatinine-ratio

**Table 4:** Correlations of blood variables between healthy dogs and cPSS-dogs before cPSS-closure.

	**Healthy dogs**			**PSS-0 dogs**		
**Correlation pairs**	**Spearman *r***	**95% CI**	***P*-value**	**Spearman *r***	**95% CI**	***P*-value**
**tCbl and TCB**	0.218	−0.129 to 0.517	.202	−0.023	−0.349 to 0.308	.892
**HCy and folate**	0.275	−0.157 to 0.619	.193	0.048	−0.280 to 0.367	.77
**HCy and albumin**	−0.029	−0.350 to 0.298	.860	0.084	−0.227 to 0.379	.588
**HCy and creatinine**	0.323	−0.001 to 0.586	.045	0.456	0.175-0.668	.002
**FA and tCbl**	n.d.	n.d.	n.d.	0.075	−0.240 to 0.375	.635
**FA and TCB**	n.d.	n.d.	n.d.	−0.034	−0.358 to 0.298	.841
**FA and HCy**	n.d.	n.d.	n.d.	−0.075	−0.375 to 0.239	.633
**FA and sMMA**	n.d.	n.d.	n.d.	−0.001	−0.334 to 0.332	.993
**FA and uMMA:crea**	n.d.	n.d.	n.d.	−0.021	−.331 to 0.293	.895

Abbreviations: CI = confidence interval; cPSS = congenital portosystemic shunt; FA = fasting ammonia; HCy = homocysteine; n.d. = not done; PSS-0 dogs = cPSS-dogs before cPSS-closure; sMMA = methylmalonic acid in serum; TCB = transcobalamin; tCbl = total cobalamin; uMMA:crea = urinary methylmalonic acid-to-creatinine-ratio.

Hypocobalaminemia was rare with cPSS, found only in 2 out of 44 (5%) PSS-0-dogs (both cIPSS; 1 puppy with normal TCB and markersB_12_met, and 1 adult dog with low TCB, normal HCy and high sMMA and uMMA:crea concentrations).

Of the normocobalaminemic PSS-0-dogs (*n* = 34/44, 77%), 11 out of 29 had low, 8 out of 29 had normal, and 10 out of 29 had high TCB concentrations. Furthermore, 1 out of 34 (3%) had high HCy concentrations, whereas hypohomocysteinemia was detected in 16 out of 34 (47%). High sMMA and uMMA:crea concentrations were detected in 17 out of 28 (61%) and 9 out of 33 (27%) of normocobalaminemic PSS-0-dogs, respectively.

Comparing PSS-0-dogs with healthy controls, there was no significant difference in tCbl (*P* = .144) and TCB concentrations (*P* = .125). However, PSS-0-dogs had significantly lower HCy (*P* < .0001) and folate (*P* < .0001) but higher sMMA (*P* < .0001) and uMMA:crea (*P* < .0001) concentrations than healthy dogs (Table 5, Figure 1). TCB concentrations > 1000 aU/L were detected in 11 out of 44 PSS-0-dogs (25%; range: 1436-10 904 aU/L) and 5 out of 39 healthy dogs (13%; range: 1381-19 411 aU/L). With the exception of 1 hypercobalaminemic PSS-0-dog, all dogs with TCB concentrations > 1000 aU/L had normocobalaminemia.

**Table 5:** Comparison of cobalamin status variables, folate, and ammonia concentrations between healthy dogs, the total group of cPSS-dogs and between cIPSS-dogs and cEPSS-dogs before cPSS-closure.

	**Healthy dogs (*n* = 39)**	**cPSS-dogs (*n* = 44)**		**cIPSS-0 (*n* = 32/44)**	**cEPSS-0 (*n* = 12/44)**	
Variable	Median (range)	Median (range)	*P*-value	Median (range)	Median (range)	*P*-value
**tCbl (pmol/L)**	479 (222-835)	601 (134-2145)	.144	537 (134-1433)	788 (223-2145)	.049
**TCB (aU/L)**	40 (40-19 411)	202 (40-10 904)	.125	447 (40-10 904)	40 (40-1436)	.019
**HCy (μmol/L)**	11.7 (6.1-24.6)	5.9 (2.5-18.3)	<.0001	5.9 (2.8-18.3)	4.8 (2.5-8.3)	.206
**sMMA (μg/L)**	85.5 (48.5-164)	175.5 (26.5-815)	<.0001	179.5 (26.5-815.0)	102.3 (44.3-255)	.049
**uMMA:crea (mg/g)**	3.0 (1.1-82.4)	22.4 (2.8-156.9)	<.0001	21.8 (2.8-156.9)	23.8 (7.0-80.2)	.715
**folate (ng/mL)**	11.1 (5.1-27.2)	6.2 (2.1-11.1)	.0001	6.3 (2.1-11.1)	5.6 (3.4-9.3)	.312
**ammonia (μmol/L)**	n.d.	933 (25-241)	n. d.	87 (25-239)	118 (64-241)	.116

Abbreviations: aU = arbitrary units; cPSS = congenital portosystemic shunt; cEPSS = congenital extrahepatic shunt; cEPSS-0 = cEPSS-dogs before cPSS-closure; cIPSS = congenital intrahepatic shunt; cIPSS-0 = cIPSS-dogs before cPSS-closure; HCy = homocysteine; n. d. = not done; PSS-0 = first study point (before cPSS-closure); PSS-12 = second study point (approximately 12 weeks after cPSS-closure); sMMA = serum methylmalonic acid; TCB = transcobalamin; tCbl = total cobalamin; uMMA:crea = urinary methylmalonic acid-to-creatinine ratio.

**Figure 1:**
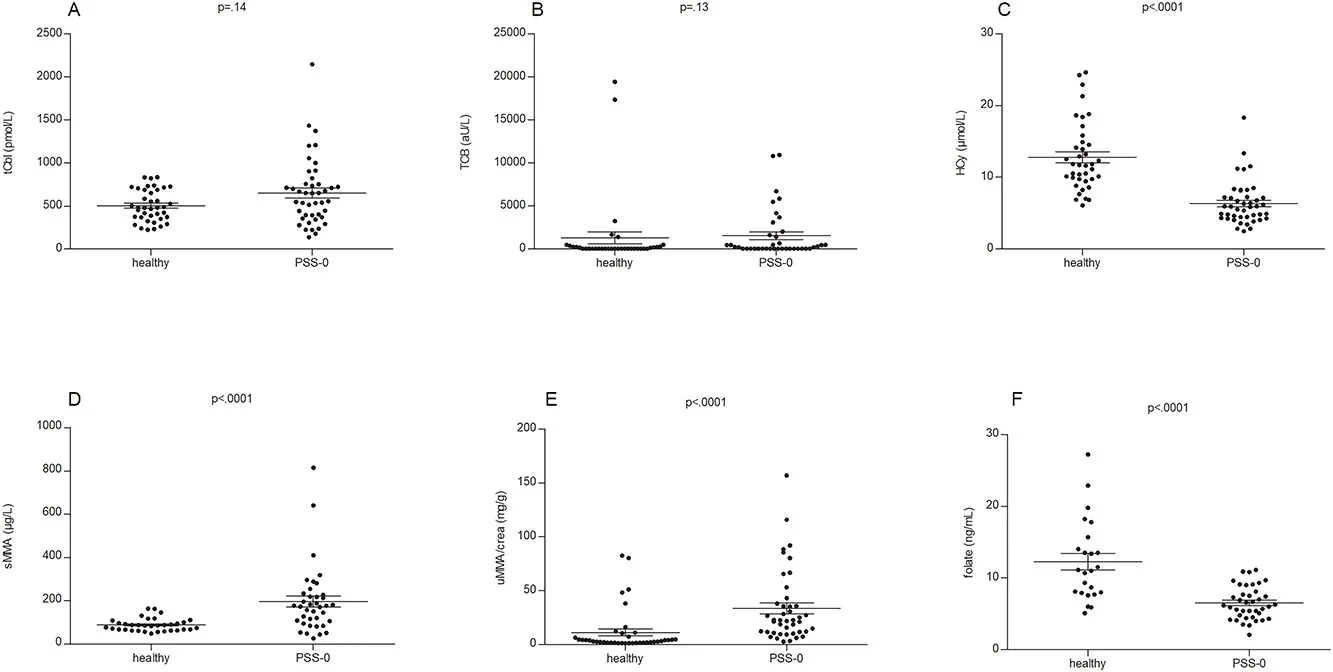
Comparison of cobalamin analyte and folate concentrations between healthy dogs and cPSS-dogs. Scatter plot of group comparison of serum total cobalamin (tCbl) (A), transcobalamin (TCB) (B), total homocysteine (HCy) (C), methylmalonic acid in serum (sMMA) (D), urine (urinary MMA-to-creatine ratio, uMMA:crea) (E), and serum folate (F) concentrations between healthy dogs and cPSS-dogs the day or a maximum of 3 days before cPSS-closure. Between both groups, there was no significant difference in tCbl and TCB concentrations but cPSS-dogs had significantly lower HCy, sMMA, uMMA:crea, and folate concentrations. Horizontal lines represent the median (central line) and the upper and lower interquartile boundary lines.

Cobalamin analytes and folate concentrations in cPSS-dogs before and after cPSS-closure.

In cIPSS-dogs, cPSS-closure was attempted by either coil (26/32, 81%) or plug embolization (6/32, 19%). Surgical cPSS-closure in cEPSS-dogs was performed by suture ligation (5/12, 42%) or thin film banding (7/12, 58%), elected at the discretion of the attending soft-tissue surgeon. Four dogs (2 each with cIPSS and cEPSS) were not re-presented for a recheck at PSS-12 due to cost or travel restrictions. Between both study points, 1 cIPSS-dog died and 1 cEPSS-dog received prolonged antimicrobial treatment for severe vomiting and diarrhea with complicating sepsis, rendering data from 38 dogs (29 cIPSS and 9 cEPSS) for PSS-12 evaluation.

At PSS-12, 3/38 (8%) dogs had hypercobalaminemia, and all 3 dogs were already hypercobalaminemic at PSS-0. Only 1 of the 3 dogs (cIPSS, incomplete cPSS-closure) had a persistently high sMMA concentration (218 mg/L; RI: 45.3-159.5^23^), while the other 2 dogs (both cEPSS, 1 complete and 1 incomplete cPSS-closure) had normal sMMA (56.1 and 49.6 μg/L) and uMMA:crea (2.8 and 4.7, RI: ≤ 22.4 mg/g^23^) concentrations. Despite normocobalaminemia, 6/31 (19%) PSS-12-dogs had high TCB concentrations (>1,000 aU/L), 2/6 also had abnormal sMMA (510 and 641 μg/L) and 1/6 abnormal (192.1 mg/g) uMMA:crea concentrations. In 5 out of 6 dogs, high TCB concentrations were detected at PSS-0. All 6 dogs had a cIPSS, with residual shunt flow at PSS-12 in 4/6.

Hypocobalaminemia was detected in 3 out of 38 (8%) PSS-12-dogs. Among these, 2 dogs were initially identified as hypocobalaminemic. The dog with concurrently suphraphysiologic sMMA (228 μg/L) and uMMA:crea (85.4 mg/g) concentrations maintained high sMMA (186 μg/L) and uMMA:crea (84.1 mg/g) concentrations at PSS-12, whereas the other dog developed an high sMMA concentration from PSS-0 (158 μg/L) to PSS-12 (304 μg/L). A third dog developed hypocobalaminemia (from 235 to 138 pmol/L, RI: 178.5-805.1 pmol/L^23^) with concurrently high sMMA (261 μg/L) but normal HCy (7.2 μmol/L; RI: 5.8-29 μmol/L^23^) and uMMA:crea (17.1 mg/g) concentrations. In all 3 dogs, cPSS-closure was assessed as incomplete.

Between both study time points, there was no significant difference in sMMA concentrations (*P* = .29). However, after cPSS-closure, cPSS-dogs had significantly lower tCbl (*P* = .0001), lower TCB (*P* = .014), higher HCy (*P* < .0001), lower uMMA:crea (*P* = .0002), and higher folate (*P* < .0001) concentrations (Figure 2) compared to the presentation at PSS-0.

**Figure 2:**
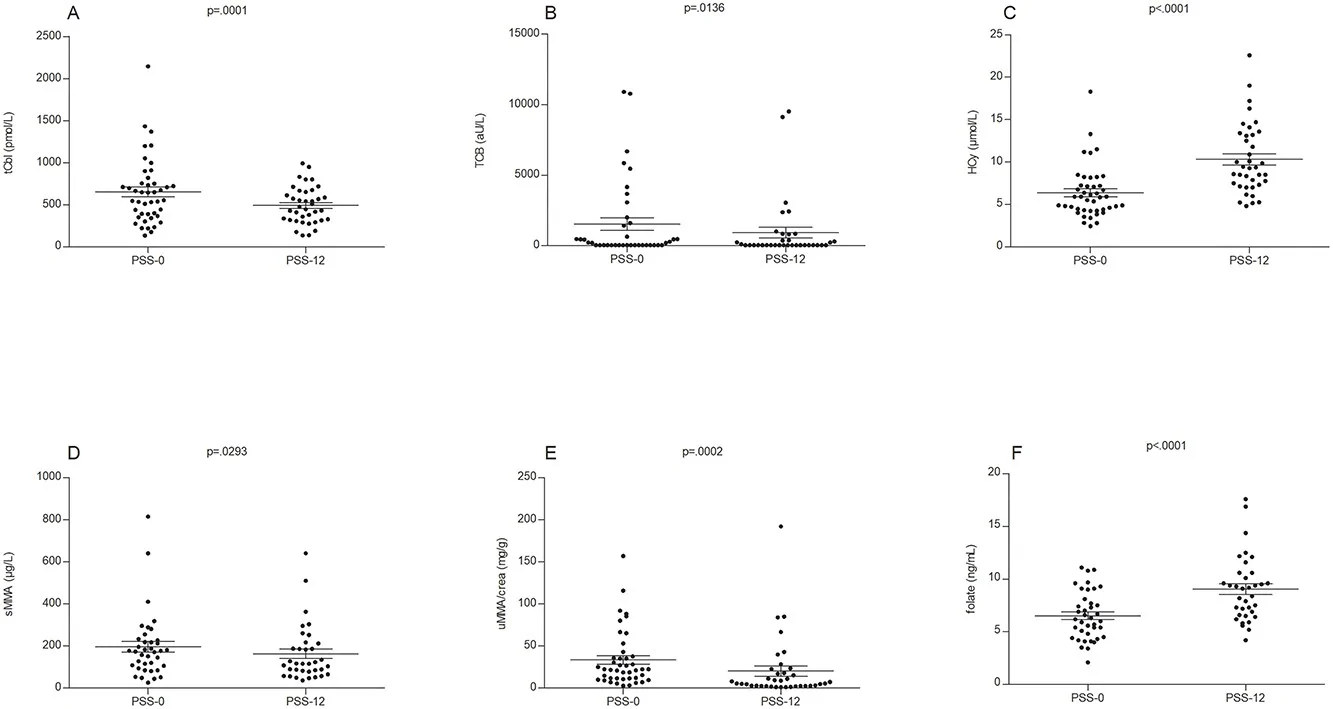
Paired comparison of cobalamin analyte and folate concentrations in cPSS-dogs before and after cPSS-closure. Scatter plot of group comparison, serum total cobalamin (tCbl) (A), transcobalamin (TCB) (B), homocysteine (HCy) (C), total methylmalonic acid in serum (sMMA) (D), urine (urinary MMA-to-creatine ratio, uMMA:crea) (E), and folate (F) concentrations between cPSS-dogs before and approximately 12 weeks after cPSS-closure. Horizontal lines represent the median (central line) and the upper and lower interquartile boundary lines. Concentrations of tCbl but not TCB lowered significantly after cPSS-closure. There was no significant difference in sMMA concentrations before and after cPSS-closure while uMMA:crea concentrations lowered significantly. In contrast, both HCy and folate concentrations rose significantly.

Cobalamin analytes and folate concentrations in cIPSS and cEPSS-dogs before and after cPSS-closure.

Before cPSS-closure, hypercobalaminemia was less common in dogs with cIPSS (3/32, 9%) than with cEPSS (5/12, 42%) and cIPSS-dogs had significantly lower tCbl concentrations than cEPSS-dogs (*P* = .049, Table 6). In contrast, cIPSS-dogs had significantly higher TCB (*P* = .019) and sMMA (*P* = .049) concentrations than cEPSS-dogs. Hypocobalaminemia was detected in 2/32 (6%) of cIPSS-dogs but was not detected in any cEPSS-dog. Among normocobalaminemic cPSS-dogs, derangements in markersB_12_met were common with either cPSS-type.

**Table 6:** Cobalamin status derangements between cIPSS-dogs and cEPSS-dogs before cPSS-closure.

**MarkersB** _**12**_ **met**	**Hypocobalaminemia**		**Normocobalaminemia**		**Hypercobalaminemia**	
**Number of dogs**	**cIPSS** **2/32**	**cEPSS** **0/12**	**cIPSS** **27/32**	**cEPSS** **7/12**	**cIPSS** **3/32**	**cEPSS** **5/12**
Number of variables with abnormal values out of available samples according to total cobalamin concentration						
**TCB** **≤ 1000 aU/L**	1/2	0	8/24	3/5	1/3	5/5
**TCB** **> 1000 aU/L**	0/2	0	9/24	1/5	1/3	0/5
**HCy ↓**	0/2	0	13/27	3/7	1/3	4/5
**HCy ↑**	0/2	0	1/27	0/7	0/3	0/5
**sMMA ↓**	0/2	0	0/24	0/7	1/3	0/5
**sMMA ↑**	1/2	0	13/24	4/7	2/3	1/5
**uMMA:crea ↑**	1/2	0	8/27	3/7	0/3	1/5

Sample specimen were not available in all normocobalaminemic and hypercobalaminemic dogs. Numbers refer to abnormal variables out of available sample specimen from a total of 32 cIPSS-dogs and 12 cEPSS-dogs, respectively.

Abbreviations: ↓ = concentration below reference interval; ↑ = concentration above reference interval; cEPSS = congenital extrahepatic shunt; cIPSS = congenital intrahepatic shunt; HCy = homocysteine; sMMA = serum methylmalonic; TCB = transcobalamin concentration; uMMA:crea = urinary methylmalonic acid-to-creatinine ratio.

At PSS-12, 29 cIPSS-dogs and 9 cEPSS-dogs were re-presented. Hypocobalaminemia was detected in 3/38 (8%) cPSS-12-dogs: 2 adults (1 cIPSS, 1 cEPSS), 1 puppy (cIPSS). Hypercobalaminemia occurred also in 3/38 (8%) cPSS-12-dogs (2 adults (1 cIPSS, 1 cEPSS), 1 puppy (cEPSS)). All hypocobalaminemic cPSS-dogs had normal HCy, high sMMA, and normal (2/3) or high (1/3) uMMA:crea concentrations. Transcobalamin and folate values were available in 2 out of 3 hypocobalaminemic cPSS-12-dogs and were physiologic. Of the hypercobalaminemic cPSS-dogs, 1 had reduced HCy, another dog had supraphysiologic sMMA and uMMA:crea concentrations. Dogs with cIPSS had significantly higher sMMA concentrations than cEPSS-dogs whereas the other variables did not differ significantly (Table 7).

**Table 7:** Comparison of cobalamin status variables, folate, and ammonia concentrations between cPSS-dogs before (PSS-0) and after cPSS-closure (PSS-12) and between cIPSS-dogs and cEPSS-dogs after cPSS closure.

	cPSS-0 (*n* = 44)	cPSS-12 (*n* = 38)		cIPSS-12 (*n* = 29)	cEPSS-12 (*n* = 9)	
Variable	Median (range)		*P*-value			*P*-value
**tCbl (pmol/L)**	601 (134-2.145)	462 (137-992)	.0001	430 (137-952)	553 (138-992)	.159
**TCB (aU/L)**	202 (40-10,904)	40 (40-9520)	.014	75.5 (40-9520)	40 (40-860)	.126
**HCy (μmol/L)**	5.9 (2.5-18.3)	9.3 (4.8-22.6)	<.0001	9.4 (4.8-22.6)	9.3 (5.3-13.6)	.891
**sMMA (μg/L)**	175.5 (26.5-815)	116 (37.4-641)	.293	131.5 (56.7-41.)	56.1 (37.4-261)	.001
**uMMA:crea (mg/g)**	22.4 (2.8-156.9)	5.6 (1.3-192.1)	.0002	9.3 (1.3-192.1)	3.1 (1.4-17.1)	.082
**folate (ng/mL)**	6.2 (2.1-11.1)	9.1 (4.2-17.6)	<.0001	9.3 (5.2-17.6)	8.4 (4.2-12.2)	.664
**ammonia (μmol/L)**	93 (25-241)	12 (.1-108)	<.0001	14 (.1-108)	7 (.1-31)	.056

Abbreviations: aU = arbitrary units; cPSS = congenital portosystemic shunt; cEPSS = congenital extrahepatic shunt; cEPSS-12 = cEPSS-dogs after cPSS-closure; cIPSS = congenital intrahepatic shunt; cIPSS-12 = cIPSS-dogs after cPSS-closure; HCy = homocysteine; n. d. = not done; PSS-0 = first study point (before cPSS-closure); PSS-12 = second study point (approximately 12 weeks after cPSS-closure); sMMA = serum methylmalonic acid; TCB = transcobalamin; tCbl = total cobalamin; uMMA:crea = urinary methylmalonic acid-to-creatinine ratio.

Comparison of cobalamin status and folate concentrations between cPSS-dogs with complete and incomplete cPSS-closure.

Complete cPSS-closure was determined in 8 out of 29 (28%) cIPSS-dogs and 6 out of 9 (67%) cEPSS-dogs. In 1 cIPSS-dog, acquired portosystemic shunts were detected at PSS-12 based on repeated ultrasonography. There was no significant difference in tCbl (*P* = .12), TCB (*P* = .5), HCy (*P* = .5), and folate (*P* = .12) concentrations between dogs with complete and incomplete cPSS-closure. In contrast, sMMA (*P* = .0001) and uMMA:crea (*P* = .003) concentrations were significantly lower in dogs with complete cPSS-closure compared with those with incomplete cPSS-closure (Figure 3).

**Figure 3:**
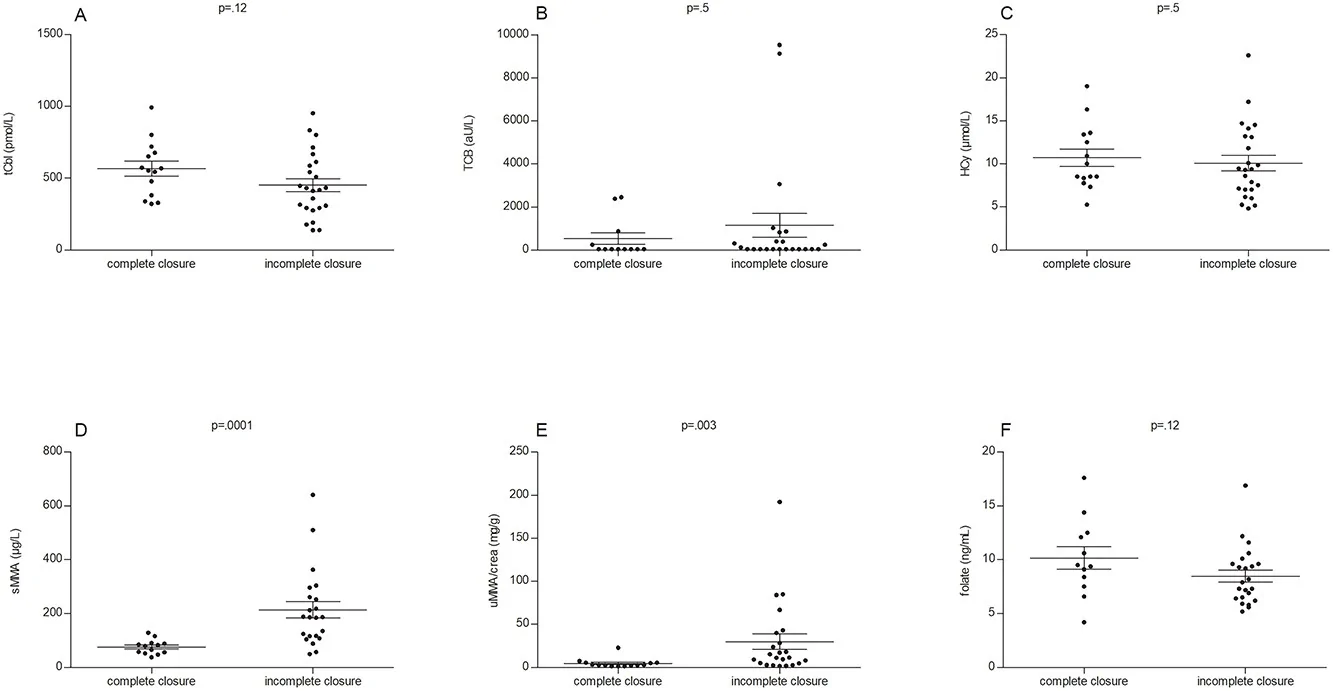
Comparison of cobalamin analyte and folate concentrations in dogs with complete and incomplete cPSS-closure. Approximately 12 weeks after their cPSS-closure, there was no significant difference regarding total cobalamin (tCbl) (A), transcobalamin (TCB) (B), homocysteine (HCy) (C), and folate (F) concentrations between dogs with complete or incomplete cPSS-closure. In dogs with incomplete cPSS-closure, serum methylmalonic (sMMA) (D) and urinary MMA-to-creatinine ratio (uMMA:crea) (E) concentrations were significantly higher. Scatter plot. Horizontal lines represent the median (central line) and the upper and lower interquartile boundary lines.

Blood ammonia and cobalamin status.

At PSS-0, 35 out of 43 (81%) dogs were hyperammonemic, with ammonia concentrations of > 60 μmol/L. Among these, 20 out of 35 (57%) dogs had ammonia concentrations > 100 μmol/L.

Of all hyperammonemic dogs, hypocobalaminemia was detected in only 1 puppy with normal HCy, sMMA, and uMMA:crea concentrations. In contrast, hypercobalaminemia occurred in 7 out of 35 (20%) hyperammonemic dogs. Four dogs (11%) had hypohomocysteinemia, 3 dogs had hyperhomocysteinemia, and 1 dog had a high uMMA:crea concentration. Normocobalaminemia was seen in 27 (77%) hyperammonemic dogs, of which 15 out of 27 (56%) and 7 out of 27 (26%) dogs had high sMMA and uMMA:crea concentrations, respectively. In 5 dogs, sMMA concentrations were unavailable.

Hyperammonemia was detected in 5 out of 38 (13%) PSS-12-dogs; 3 out of 5 dogs had ammonia concentrations > 100 μmol/L. All hyperammonemic PSS-12-dogs had a cIPSS with incomplete cPSS-closure. In these 5 dogs, tCbl concentrations were within the normal RI, but 4 dogs had supraphysiologic sMMA concentrations (sMMA was not available from the remaining dog). PSS-12 uMMA:crea concentrations were available from 4 dogs and were high in 1 out of 4. Hypohomocysteinemia occurred in only 1 out of 5 dogs.

Laboratory variables with potential associations with cobalamin analyte levels.

Blood albumin and creatinine concentrations were significantly (*P* < .0001) higher in healthy dogs than in PSS-0-dogs whereas CRP concentrations were significantly lower (*P* = .027). In PSS-0-dogs, there was a moderate positive correlation between HCy and blood creatinine concentrations. Bacterial culture was performed in 38 PSS-0-dogs and revealed significant bacterial growth in 3 out of 38 (8%) samples whereas all bacterial cultures available in 30/39 healthy dogs tested negative (Table 8).

**Table 8:** Laboratory variables affecting cobalamin status variables. Variables were compared between healthy dogs and cPSS-dogs before cPSS-closure.

	**Healthy dogs**	**cPSS-dogs**		
**Variable**	**Median** (range)	**Median** (range)	***P*-value**	**Comment**
**Serum albumin (RI: 26.2-34.4 g/L)**	30.2 (24.5-34.2)	24.6 (16.2-29.0)	<.0001	
**Serum creatinine (RI: 53-122 μmol/L)**	86.7 (52.2-140.1)	44.6 (16.7-81.8)	<.0001	1 healthy dog with creatinine concentration > 122 μmol/L had a corresponding USG > 1.050 and sonographically unremarkable kidneys; thus, this dog was considered healthy (possibly prerenal azotemia)
**Serum CRP (RI: 0-10.8 mg/L)**	1.8 (0-10.9)	2.6 (0.1-40.2)	.027	
**USG**	1.046 (1.014-1.060)	1.018 (1.003-1.060)	<.0001	
**Urine bacterial culture**	available in 30/39 dogs; 30/30 dogs negative	available in 38/44 dogs; 3/38 dogs positive (2 positive for *E.coli*, 1 positive for *E.coli* and *Enterococcus faecium*)	n.a.	1 PSS-0 dog with bacteriuria had increased uMMA:crea concentrations concurrently with increased sMMA but normal HCy concentration. In all dogs without bacterial cultures, uMMA:crea concentrations were within the normal RI.

Abbreviations: CRP = C-reactive protein; HCy = homocysteine concentration; cPSS = congenital portosystemic shunt; n.a = not applicable; PSS-0 = before cPSS-closure; RI = reference interval; sMMA = serum methylmalonic acid; uMMA:crea = urinary methylmalonic acid-to-creatinine ratio; USG = urine specific gravity.

Comparing PSS-0 and PSS-12-dogs, blood albumin and creatinine concentrations were significantly higher after cPSS-closure (both *P* < .0001). Albumin concentrations rose to a median of 27.6 g/L (range: 24.3-39.5 g/L) and creatinine concentrations to a median of 61.4 μmol/L (range: 41.0-147.0 μmol/L).

## Discussion

The results of this study showed that abnormalities of cobalamin, TCB, and markers of B_12_metabolism were common in cPSS-dogs. Besides a high proportion of cPSS-dogs with hypercobalaminemia, there was a large number of normocobalaminemic dogs with changes in cobalamin status variables suggestive of a functional (ie, intracellular) cobalamin deficiency. Alterations ameliorated within 3 months after cPSS-closure, however, in a small proportion of cPSS-12-dogs cobalamin deficiency (hypocobalaminemia along high markersB_12_met) remained and functional cobalamin deficiency was still present in a quarter of normocobalaminemic cPSS-dogs. Interestingly, cobalamin status derangements were not associated with hyperammonemia. Dogs with cIPSS and cEPSS differed in their cobalamin status, and hypercobalaminemia was more common with cEPSS.

Albeit a frequent finding, hypercobalaminemia has long been considered clinically irrelevant. However, in people, hypercobalaminemia without prior supplementation is associated with liver disease, cancer, and alcoholism.^17^ In dogs, hypercobalaminemia is described with gastrointestinal, hepatic, neoplastic, and renal disease.^15^^,^^16^^,^^28^ In some hypercobalaminemic people, functional cobalamin deficiency (high markersB_12_met despite normal or supraphysiologic tCbl concentrations) occurs^24^^,^^29^^,^^30^ with some even showing clinical signs or biochemical changes characteristic of hypocobalaminemia (eg, tiredness, cognitive dysfunction, spastic paraparesis, skin lesions, atrophic glossitis, and macrocytosis, megaloblastic anemia, and neutrophilic hypersegmentation).^17^^,^^31–34^ Besides excessive supplementation, hypercobalaminemia can be caused by higher release from storage tissue, higher production or reduced clearance of haptocorrins, or deficiency or reduced affinity to TCB.^17^^,^^28^^,^^35^ Recently, the prevalence of hypercobalaminemia was described as 90% in dogs with cEPSS, which persisted in 70% of the dogs for 3 months after complete cPSS-closure.^14^ In our cPSS study group, the overall prevalence of hypercobalaminemia (8/44, 18%) before cPSS-closure was much lower. Most likely, this discrepancy was caused by the inclusion of both cPSS-types as the former study included cEPSS only whereas the predominant cPSS-type here was cIPSS. Hypercobalaminemia was less prevalent in our cIPSS-dogs compared with cEPSS-dogs and cEPSS-dogs had significantly higher tCbl concentrations. Between cIPSS and cEPSS, differences in laboratory variables are documented.^36^ This might reflect different grades of severity of the underlying different cPSS forms which seem to remain even after cPSS-closure because cIPSS-dogs had significantly higher sMMA concentrations compared with cEPSS-dogs. However, the other variables did not differ significantly. Yet, because of the small number of cEPSS-dogs in our study, results need to be assessed cautiously, especially those after cPSS-closure. Even so, a high prevalence of hypercobalaminemia with cEPSS was also previously published^14^ and thus, our findings seem plausible. The underlying cause of hypercobalaminemia in cPSS remains unclear. Potential causes include prolonged cobalamin circulation times due to hepatic bypass or reduced clearance/degradation of haptocorrin.^17^^,^^37^ Cellular cobalamin uptake requires the presence of the TCB receptor CD320 protein.^37–39^ Hepatocytes appear to lack this receptor,^17^^,^^37^ and cobalamin is likely predominantly extracted from the blood by endothelial cells which subsequently transfer cobalamin to hepatocytes.^40^ Due to vascular malformation and hepatic hypoperfusion,^41–43^ cPSS-dogs might have a reduction in hepatic endothelial cell mass or function, potentially leading to a reduced cobalamin uptake and storage. Additionally, while TCB is predominantly produced by the liver, haptocorrin is mainly formed by granulocytes,^17^^,^^37^^,^^44^ and higher amounts of granulocyte-derived haptocorrin could contribute to hypercobalaminemia as inflammation and oxidative stress are common in dogs with cPSS.^45^^,^^46^

Total cobalamin concentrations significantly lowered between PSS-0 and PSS-12, but did not differ between dogs with and without complete cPSS-closure. Possibly, the improved or normalized nutrient-rich blood flow to the liver might have caused an enhanced extraction of circulating cobalamin by the liver or a higher clearance of haptocorrin.^37^ Despite statistical differences between PSS-0 and PSS-12-dogs considerable overlap was detected regarding the concentrations of cobalamin status variables and folate concentrations which was also seen in cPSS-12-dogs with complete and incomplete cPSS-closure regarding sMMA and uMMA:crea concentrations. Also, less though considerable overlap existed between markersB_12_met and folate concentrations comparing healthy dogs and PSS-0-dogs. Intuitively, a more pronounced separation of these variables between healthy dogs and cPSS-dogs and therefore also between cPSS-dogs before and after cPSS-closure would be expected, especially when complete cPSS-closure was achieved. Differences in hepatic perfusion and thus liver function between both healthy dogs and cPSS-dogs and cPSS-dogs before and after (complete) cPSS-closure would be assumed to result in clearer differences regarding hepatic perfusion associated liver function and thus, vitamin storage, distribution and availability to cells. However, persistent metabolic alterations including hypercobalaminemia,^14^ alterations in amino acid concentrations^47^ and high SBAs^48^ after complete cPSS-closure are recognized, reflecting a prolonged recovery of liver function even after complete cPSS-closure. Thus, overlap between cobalamin status variables and folate in cPSS-dogs before and after cPSS-closure are likely caused by persisting alterations of liver function.

In people, due to its higher specificity, determination of the active form of cobalamin, holo-transcobalamin (cobalamin bound to TCB), is often preferred over tCbl concentrations as holo-transcobalamin comprises only 20%-30% of tCbl.^15^^,^^17–19^^,^^49^ While holo-transcobalamin is unavailable for dogs, measuring its binding protein, TCB, might be a suitable proxy for indirect holo-transcobalamin determination. Yet, in both healthy and PSS-0-dogs, tCbl and TCB concentration did not correlate, which could reflect an effect of the predominant and potentially variable haptocorrin bound cobalamin concentration on tCbl levels.^15^ In 18 out of 39 (46%) of PSS-0-dogs, TCB concentrations were below the detection limit, possibly reflecting diminished production because of hepatic dysfunction.^28^ However, TCB concentrations were also undetectable in 21 out of 36 (58%) of healthy dogs with normal liver function. In contrast, very high TCB concentrations were found in 5 out of 36 (14%) healthy dogs and 6 out of 39 (15%) PSS-0-dogs, but only 1 of these dogs (cIPSS) had hypercobalaminemia. Hence, even very high TCB concentrations only have a minor effect on tCbl concentration, likely because of the small proportion of active cobalamin. High concentrations of TCB have been associated with renal and hepatic disease,^28^^,^^50^ but are detected in some people who are unable to utilize cobalamin properly. Reduced cobalamin availability or utilization in cPSS-dogs could occur due to liver bypass and hepatic dysfunction and thus cause high TCB concentrations.

Mammals depend on the consumption of cobalamin, primarily by intake of food products of animal origin.^51^ After gastric release and intestinal uptake, cobalamin is transported to the liver, its major storage site.^37^ Cobalamin is already stored in the fetal liver as the fetus receives the majority of the mother’s blood cobalamin content.^52–54^ Thus, liver bypass could early affect cobalamin storage in the fetal liver. Reduced cobalamin availability in affected dogs might be further exacerbated by the persistent liver bypass in combination with dietary meat restriction to ameliorate hyperammonemia-associated hepatic encephalopathy.^12^^,^^55^^,^^56^

With cobalamin deficiency at the cellular level, concurrent hyperhomocysteinemia would be expected as cobalamin also functions as an essential cofactor of the enzyme methionine synthase.^1^^,^^49^^,^^57^^,^^58^ However, cPSS-dogs had significantly lower HCy concentrations than healthy dogs. Circulating HCy is mostly bound to albumin^59^ and blood albumin concentrations could affect total HCy blood concentrations. Actually, albumin concentrations in cPSS-dogs were significantly lower than in controls. Although hypoalbuminemia theoretically could explain lower HCy concentrations in our cPSS-dogs, there was no correlation between HCy and albumin concentrations. Within the body, HCy is formed from methionine, which predominantly originates from meat protein.^60^ To prevent hyperammonemia-associated hepatic encephalopathy,^13^ cPSS-dogs received a protein-restricted liver diet.^12^^,^^55^^,^^61^ Therefore, low HCy concentrations could be a nutritional consequence due to methionine deficiency resulting from feeding a protein-restricted diet. Additionally, even though a possible higher predisposition for cIPSS-dogs, cPSS-dogs generally are at risk for gastrointestinal ulceration before and after cPSS-closure.^62–65^ Hence, administration of a proton-pump inhibitor is part of the treatment cPSS-dogs in our clinic, and all cPSS-dogs received omeprazole for a minimum of 4 weeks. Supraphysiologic gastric pH might affect protein digestion,^66^ possibly aggravating the already reduced supply of methionine and promoting hypohomocysteinemia. Furthermore, HCy provides about 50% of the intracellular glutathione concentration. During oxidative stress, higher HCy influx into the transsulfuration pathway indirectly leads to higher glutathione production.^67^ As high oxidative stress exists in dogs with cPSS,^45^ oxidative stress and, in consequence, a higher demand for glutathione could also reduce plasma HCy concentration.^68^^,^^69^ Homocysteine concentrations rose after cPSS-closure. Possibly because of a reduction of oxidative stress or inflammation. In children, HCy concentrations increase with age^70–72^ and are positively correlated with body mass index, muscle mass, and creatinine concentrations.^73^^,^^74^ Increasing muscle mass by increasing age and improved utilization of nutrients after cPSS-closure are other explanations for the HCy increase in PSS-12-dogs, as there was a moderate correlation between HCy and blood creatinine concentrations. This could have been further enhanced by dietary changes, as (though against recommendation) some dog owners changed their dog’s diet to a more meat-based diet soon after cPSS-closure, especially where complete cPSS-closure was achieved. Concerning changes in HCy and possible dietary effects, the significant increase in serum folate concentrations after cPSS-closure was unexpected. Folate is mainly found in green vegetables and legumes^75^ and higher concentrations would rather have been expected in PSS-0-dogs when fed higher amounts of legumes as a meat protein substitute. In people, other major causes for hyperfolatemia comprise reduced glomerular filtration rate,^76^ “trapped” folate because of underlying cobalamin deficiency,^77^ and artifacts such as hemolyzed blood samples,^78^ all unlikely in our study population. In contrast, intestinal dysbiosis, an important cause of hyperfolatemia,^79^^,^^80^ is common in cPSS-dogs^81^ and would be a reasonable explanation for hyperfolatemia in our cPSS-dogs. However, cPSS-closure would be expected to ameliorate dysbiosis and thus, reduce folate concentrations. As such, the concurrent increase in Hcy and folate concentrations at PSS-12 cannot be explained.

Although hypocobalaminemia was rare among cPSS-dogs, cobalamin status derangements suggestive of a functional cobalamin deficiency were common. Cobalamin deficiency at the cellular level might be masked by normo- or hypercobalaminemia due to high concentrations of circulating haptocorrin. Cobalamin is essential for the activity of methylmalonyl-CoA mutase,^1^^,^^49^^,^^57^ a key enzyme for the degradation of amino and odd-chain fatty acids that are ultimately metabolized to methylmalonyl-CoA. Cobalamin deficiency impairing the activity of this enzyme can consequently cause increases of MMA.^19^^,^^57^^,^^82^ Another cause for supraphysiologic MMA concentrations is renal insufficiency,^83–85^ but there was no suspicion of renal disease in any of the cPSS-dogs in our study. Urinary bacterial bioformation was the suspected cause for a high uMMA:crea concentration in a dog with Imerslund-Graesbeck syndrome (a genetic disorder causing malfunction of the intestinal cubam receptor leading to reduced cobalamin absorption and thus, hypocobalaminemia^8^), as treatment of this dog’s bacterial urinary tract infection was followed by a lowering in uMMA:crea concentration.^86^ However, bacteriuria was rare in our cPSS-dogs. In contrast, intestinal dysbiosis is commonly seen in medically treated cPSS-dogs.^81^ Dysbiosis can cause higher cobalamin consumption and rises in MMA concentrations.^87–89^ Thus, intestinal dysbiosis could have contributed to varying MMA concentrations in our study. As intestinal microbiota analysis was not performed, the data do not allow to assess a possible effect of intestinal dysbiosis on MMA concentrations. While uMMA:crea concentrations significantly declined after cPSS-closure, sMMA concentrations did not. This was unexpected because MMA concentrations in blood and urine are expected to follow simultaneous trends,^90^ provided that the glomerular filtration rate (GFR) remains unaltered^83^^,^^91^ and the patient is not a neutered male dog.^23^ Closure of a cPSS causes GFR reduction^92^ and might affect MMA excretion. However, GFR should return to normal and thus, GFR reduction seems less likely to be the cause for the opposing trends in MMA levels that cannot be explained by the results or our study. Interestingly, between cPSS-dogs with and without complete cPSS-closure, concentrations of sMMA and uMMA:crea differed significantly and both variables could potentially assist with the evaluation of the effectiveness of cPSS-closure where diagnostic imaging modalities are not readily available or yield ambiguous results.

Hyperammonemia is a common finding in cPSS-dogs and 1 of the most important contributors to hepatic encephalopathy.^13^ Cobalamin deficiency can indirectly contribute to hyperammonemia because high methylmalonyl-CoA concentrations can inhibit the carbamoyl phosphate synthetase,^11^ which catalyzes the conversion of ammonia to carbamoyl phosphate and initiates the urea cycle.^10^^,^^93^ Before cPSS-closure, most PSS-0-dogs were hyperammonemic, but neither tCbl nor TCB or sMMA concentrations were correlated with FA concentrations. Hence, despite common derangements in cobalamin analytes or markersB_12_met in this cPSS study population, cobalamin status derangements do not seem to be a major contributor to hyperammonemia in cPSS-dogs.

One important limitation of our study is that the assessment of cPSS-closure at PSS-12 was based solely on abdominal ultrasonography. At our clinic, computed tomography (or scintigraphy) is not part of the routine examination for cPSS-closure evaluation. However, ultrasonographic evaluation is regarded widely operator-dependent and inferior to computed tomography or scintigraphy.^94^^,^^95^ Furthermore, artifacts by cPSS-closure devices could disguise the presence of acquired PSS and thus, multiple acquired PSS could have been missed causing misinterpretation of complete cPSS-closure.^96^ Nevertheless, the data reported on ultrasonography performance refer primarily to the new diagnosis of a cPSS rather than cPSS-closure. To our knowledge, no data exist for evaluating cPSS-closure based on ultrasonography in comparison to other imaging modalities and no study has been published regarding the effect of cPSS closing devices on the diagnosis of acquired PSS. Furthermore, the sensitivity of ultrasonography-based cPSS detection can be as high as 92%.^97^ In addition, combined evaluation of ammonia concentration with pre- or post-prandial bile acid concentrations has a sensitivity of 95-96.3%.^98^ Thus, abdominal ultrasonography by an experienced radiologist in combination with ammonia and SBA testing appears to be a reasonable approach to allocate dogs based on ultrasonography as complete vs. incomplete cPSS-closure at PSS-12. The distribution of cIPSS and cEPSS dogs was largely uneven with a predominance of cIPSS-dogs included. While at PSS-0 the number of cEPSS dogs available for comparison of cobalamin analytes between the 2 cPSS-types was already small, this number was even smaller at PSS-12 with only 6 and 3 cEPSS-dogs left for assessment of complete and incomplete cPSS-closure. However, significant differences were evident in this small cEPSS study group, and further studies might help to understand clinical differences in cPSS-types that could lead to treatment recommendations tailored to the individual cPSS form. Additionally, there was a large proportion of healthy and cPSS-dogs with TCB concentrations below the detection limit of the assay, causing the need for left censoring of the data to adjust for statistical analysis. This could have biased the results of our study, and further studies are needed to evaluate whether TCB measurements are valuable as an additional markeB_12_met to assess the cobalamin status in dogs.

The data of this study do not allow for assessment of the influence of (dietary) methionine on HCy concentrations. Methionine concentrations are highest in animal-derived foods, especially meat and fish.^99^ Although the majority of cPSS-dogs were fed a commercial hepatic (protein-restricted) therapeutic diet, many dogs additionally received variable amounts of dairy or vegetable protein that also (to a lower extent) contain methionine.^99^ In addition, between both study time points, some owners changed their dogs’ diet without prior veterinary consultation and opted for diets with higher meat protein contents. This occurred predominantly with dogs that had evidence of complete cPSS-closure at the time of discharge after the closing procedure. Therefore, nutritional effects on methionine concentrations and consequently on HCy concentrations remain unclear. Another important limitation is that, for animal welfare reasons, liver biopsy specimens were neither available in cPSS nor healthy dogs. Hence, neither the quantification of cobalamin in hepatic tissues nor the evaluation of cobalamin surface cell receptors was possible. Thus, potential differences in cobalamin storage between cPSS and healthy dogs as a cause of either functional cobalamin deficiency or hypercobalaminemia in dogs with cPSS remain unknown. Further, data interpretation of vitamin status derangements between healthy dogs and cPSS-dogs as well as cPSS-dogs before and after cPSS-closure need to be interpreted in the light of overlapping concentrations between groups.

We conclude that data from this study suggest that cobalamin status derangements are a common finding in cPSS-dogs. The presence of a functional cobalamin deficiency is common even in cases with normocobalaminemia, and functional cobalamin deficiency remains in a substantial proportion of dogs 3 months after cPSS-closure. As tCbl concentrations are unreliable for evaluating cellular cobalamin availability, additional use of markersB_12_met should be considered. In terms of cPSS-closure evaluation, sMMA and uMMA:crea concentrations might further help to assess the completeness of cPSS-closure.

## Off-label antimicrobial declaration

Authors declare no off-label use of antimicrobials.

## Institutional animal care and use committee or other approval declaration

Approved by the local ethics committee of the Justus Liebig university of Giessen(V 54 – 19 c 20 15 h 02 GI 18/17 kTV 15/2020). Both dogs with cPSS and healthy controls were privately owned dogs that were enrolled after receiving informed written owner consent.

## Appendix

### Material and methods

This prospective study was approved by the local ethics committee (V 54 – 19 c 20 15 h 02 GI 18/17 kTV 15/2020). Both dogs with cPSS and healthy dogs were privately owned dogs that were enrolled after receiving informed written owner consent.

### Dogs with congenital portosystemic shunt

The diagnosis of a cPSS included an abnormal SBA concentration determined via SBA stimulation test and an abnormal blood ammonia concentration performed as either “basal” ammonia concentration if ammonia concentration was > 100 μmol/L or ammonia tolerance test in dogs with blood ammonia concentrations < 100 μmol/L. Additionally, the presence of a hepatic macrovascular abnormality had to be confirmed by diagnostic imaging (abdominal ultrasound or computed tomography, or both). After a minimum of 4 weeks of medical stabilization, including liver diet (with or without the addition of vegetables or milk/plant-based proteins), oral lactulose, and proton pump inhibitors, all cPSS-dogs were presented for a cPSS-closure procedure (first study time point, PSS-0). Before the procedure, dog owners had to complete a standardized questionnaire regarding their dog’s general health and wellbeing (eg, animal origin, current husbandry; feeding modalities (type and duration of diet and addition of protein sources, minerals, vitamins; vaccination and deworming; concurrent diseases). Specific questions related to the cPSS comprised occurrence, type, and frequency of neurological signs (eg, seizures, behavioral abnormalities, stupor, coma); gastrointestinal signs (eg, diarrhea, vomiting, hyporexia, hypersalivation, weight loss/insufficient weight gain, borborygmi, melena); urinary tract signs (eg, signs of cystitis, pigmenturia, urine retention), and previous medical cPSS treatment).

Exclusion criteria were refusal of cPSS-closure by the owner, the presence of comorbidities, vegan diet, vitamin supplementation, and antimicrobial treatment within the 3 months before cPSS-closure and until the re-check.

To assess the degree of cPSS-closure and the postprocedural disease course, cPSS-dogs were re-evaluated approximately 12 weeks after cPSS-closure (second study point, PSS-12), at which the owners provided follow-up information by completing the study questionnaire again. At PSS-12, all dogs underwent an overall health check including a thorough physical examination, basic laboratory data (complete blood count, biochemistry including C-reactive protein, and urinalysis with bacterial culture), SBA stimulation test, ammonia testing, and abdominal ultrasound, including color Doppler flow measurements to assess residual shunt flow.

### Healthy control dogs

Healthy dogs of any breed, aged between 2 months and 10 years, were recruited, chosen to match cPSS-dogs by age and body weight. Eligibility for study participation was assessed by a questionnaire including general questions related to the dogs’ housing and wellness (eg, animal origin, husbandry, feeding modalities, travel history, vaccination, and deworming status), and specific questions regarding the dogs’ health, focusing on possible underlying chronic diseases such as neurological, gastrointestinal, renal, dermatologic, neoplastic, or cardiovascular disease as described previously.^23^ Overall health was assessed by a thorough physical examination and basic laboratory data (complete blood count, biochemistry, activity of 1,2-o-dilauryl-rac-glycero-3-glutaric acid lipase (DGGR-lipase), C-reactive protein, and urinalysis including bacterial culture if samples were collected by cystocentesis or urinary catheter). Abnormalities of the physical examination (eg, heart murmur, BCS < 4 and ≥ 7, subcutaneous or cutaneous nodules, skin lesions), basic laboratory data (eg, anemia, increased C-reactive protein, decreased protein concentration, azotemia), or responses in the questionnaire raising the suspicion of an underlying disease (eg, “sensitive stomach,” soft stools, vomiting, weight loss, pruritus, polyuria/polydipsia, voiding abnormalities, medications given within the last 12 months other than antiparasitic treatment, previous blood work abnormalities) or receiving a vegan diet, or both, vitamin supplementation or systemic antimicrobial treatment within the last 3 months (puppies) or 6 months (adult dogs) before study entry led to exclusion.

### Blood samples

Before sampling, food was withheld for at least 8 h (all cPSS-dogs and healthy puppies < 1 year) or 12 h (healthy adult dogs). In cPSS-dogs, sampling was performed either the day of the cPSS-closure or within a maximum of 3 days before the procedure, depending on the schedule of the cPSS-closure.

Blood was collected directly into plain (SBA, tCbl, TCB, serum methylmalonic acid (sMMA), and folate testing), heparin (biochemistry), sodium fluoride (homocysteine (HCy) measurement), and tripotassium ethylenediamine tetraacetic acid (EDTA) tubes (hematology), in the specified order.

Basic laboratory examination comprised hematology (ADVIA2120/2120i, Siemes Healthcare GmbH, Erlangen, Germany) and biochemistry panels (ABX Pentra C400, ABX Horiba, Montpellier, France) including the activity of 1,2-o-dilauryl-rac-glycero-3-gludaric acid lipase (DGGR lipase) and concentrations of C-reactive protein (Canine CRP, Gentian AS, Moss, Norway; performed on ABX Pentra C400, ABX Horiba, Montpellier, France) (CRP). In cPSS-dogs, 20 μL of whole blood were used to measure blood ammonia concentration using a point-of-care blood ammonia device (PocketChem BA PA-4140, Arkray Europe, B.V., Amstelveen, The Netherlands).

### Urine samples

In cPSS-dogs, all samples were collected by cystocentesis whereas in healthy dogs, samples were acquired by cystocentesis, transurethral catheterization, or free-catch (Refractometer (handheld), Protein 0-12 g/dl, ATC, Euromex Holland, Arnhem, The Netherlands). Urinalysis included urine specific gravity (USG) (Combur® 9 Test, Roche Diagnostics (Schweiz) AG, Rotkreuz, Switzerland), dip stick analysis (GraphPad Prism v5, GraphPad Statistical Software, Boston, Massachusetts), and manual sediment evaluation. In PSS-0-dogs and healthy dogs, bacterial culture was performed in samples collected via cystocentesis or catheterization provided sufficient sample volume was available.

### Measurement of cobalamin status variables and folate concentration

Concentrations of tCbl, TCB, HCy, sMMA, uMMA:crea (urinary MMA, normalized against urinary creatinine), and folate were determined at both study points (PSS-0 and PSS-12) in cPSS-dogs and at a single time point in the healthy dogs.

Total cobalamin and folate concentrations were analyzed by a solid-phase, competitive chemiluminescent enzyme immunoassay (IMMULITE^®^ 2000) as published.^23–27^ A validated liquid chromatography/tandem mass spectrometry (LC–MS/MS) assay was used to assess the markersB_12_met (HCy, sMMA, uMMA:crea). The LC–MS/MS method used, handling of study specimens, and RI determined from healthy dogs were previously described.^23^

### Measurement of serum transcobalamin

Serum TCB concentrations were measured by a validated in-house direct sandwich enzyme-linked immunosorbent assay as previously described.^20^ The assay utilized seven calibrators (human serum with known holo-transcobalamin concentration serially diluted 1:5-1:320 in assay buffer). Affinity-purified anti-TCB antibodies were used for capturing and, after biotin conjugation, for detection. After incubation with avidin-HRP (horseradish peroxidase), an HRP substrate was used for color development, and a 5-variable logistic curve fit served to generate the standard curve. The lower detection limit of the assay for serum samples from dogs is 80 arbitrary units (aU)/L. Samples were analyzed over a total of 4 consecutive assay runs, using the same reagent lots, standards, and quality controls.

### Statistical analysis

Statistical analyses were performed using GraphPad Prism v5 (GraphPad Statistical Software, Boston, MA) Distribution of data normality was assessed by a Shapiro–Wilk test.

To assess possible differences between groups, the following comparisons were made^1^: assessment of cobalamin status (tCbl, TCB, markersB_12_met) and folate concentrations between PSS-0 and healthy dogs^2^; comparison of changes of cobalamin status and folate concentrations between PSS-0-dogs and PSS-12-dogs^3^; evaluation of differences between cIPSS-dogs and\textcolor[rgb]{0,0,0}{text} cEPSS-dogs before and after cPSS-closure^4^; comparison of differences between cPSS-dogs with complete and incomplete cPSS-closure, overall and between both cPSS-types; and^5^ evaluation of associations between ammonia and cobalamin status.

Independent 2-group comparisons of tCbl, TCB, HCy, sMMA, uMMA:crea, and folate concentrations between cPSS-dogs and healthy dogs were performed by a Mann–Whitney-*U* test. Blood albumin and creatinine concentrations were compared using a parametric unpaired *t-*test, whereas USG was evaluated by a Mann–Whitney-*U* test. For TCB, samples with TCB concentrations below the detection limit of the assay (<80 aU/L) were left censored. Censored values were replaced with values equal to half the detection limit (40 aU/L).

At PSS-0, possible correlations between tCbl and TCB, between HCy and folate, albumin or creatinine and between ammonia and tCbl, TCB, HCy, sMMA, or uMMA:crea concentrations were assessed by Spearman’s correlation.

At PSS-0, comparisons of cobalamin status and folate concentrations between cIPSS-dogs and cEPSS-dogs were analyzed by a Mann–Whitney-*U* test and an unpaired *t-*test, respectively. Cobalamin analytes, folate, blood albumin, and creatinine concentrations between PSS-0 and PSS-12 were compared using a Wilcoxon matched-pairs (signed rank) test. With the exception of tCbl concentrations (unpaired *t-*test), comparison of TCB, markersB_12_met, and folate between dogs with complete vs. incomplete cPSS closure at PSS-12 was performed by a Mann–Whitney-*U* test. For all test comparisons, the level of significance was set at *P* < .05.

## Abbreviations

CI: confidence interval
CLIA: chemiluminescence immunoassay
CFU: colony-forming unit; cPSS congenital portosystemic shunt
cEPSS: congenital extrahepatic portosystemic shunt; congenital intrahepatic portosystemic shunt
CRP: C-reactive protein; DGGR-lipase—activity of 1,2-o-dilauryl-rac-glycero-3-glutaric acid lipase
DNA: deoxyribonucleic acid
EDTA: ethylenediaminetetraacetic acid
HCy: homocysteine, measured as total homocysteine concentration
LC–MS/MS: liquid chromatography/tandem mass spectrometry
markersB_12_met: markers of cobalamin metabolism
RI: reference interval
sMMA: methylmalonic acid in serum
TCB: transcobalamin
tCbl: total cobalamin
uMMA:crea: methylmalonic acid in urine, given as the ratio to urinary creatinine
USG: urine specific gravity

## References

[1] Allen RH, Stabler SP, Savage DG, Lindenbaum J. Metabolic abnormalities in cobalamin (vitamin B12) and folate deficiency. FASEB J. 1993;7:1344-1353. 10.1096/fasebj.7.14.7901104 7901104

[2] Bailey LB, Stover PJ, McNulty H, et al. Biomarkers of nutrition for development-folate review. J Nutr. 2015;145:1636s-1680s. 10.3945/jn.114.206599 PMC4478945 26451605

[3] Ruaux CG . Cobalamin in companion animals: diagnostic marker, deficiency states and therapeutic implications. Vet J. 2013;196:145-152. 10.1016/j.tvjl.2013.01.025 23518469

[4] Allen LH, Miller JW, de Groot L, et al. Biomarkers of nutrition for development (BOND): vitamin B-12 review. J Nutr. 2018;148:1995s-2027s. 10.1093/jn/nxy201 PMC6297555 30500928

[5] Hörster F, Hoffmann GF. Pathophysiology, diagnosis, and treatment of methylmalonic aciduria-recent advances and new challenges. Pediatr Nephrol. 2004;19:1071-1074. 10.1007/s00467-004-1572-3 15293040

[6] Fordyce HH, Callan MB, Giger U. Persistent cobalamin deficiency causing failure to thrive in a juvenile beagle. J Small Anim Pract. 2000;41:407-410. 10.1111/j.1748-5827.2000.tb03233.x 11023127

[7] Gold AJ, Scott MA, Fyfe JC. Failure to thrive and life-threatening complications due to inherited selective cobalamin malabsorption effectively managed in a juvenile Australian shepherd dog. Can Vet J. 2015;56:1029-1034. PMC4572816 26483576

[8] Fyfe JC, Hemker SL, Venta PJ, Stebbing B, Giger U. Selective intestinal cobalamin malabsorption with proteinuria (Imerslund-Gräsbeck syndrome) in juvenile beagles. J Vet Intern Med. 2014;28:356-362. 10.1111/jvim.12284 PMC3959579 24433284

[9] Sauer H, Wilmanns W. Cobalamin dependent methionine synthesis and methyl-folate-trap in human vitamin B12 deficiency. Br J Haematol. 1977;36:189-198. 10.1111/j.1365-2141.1977.tb00639.x 871432

[10] Dimski DS . Ammonia metabolism and the urea cycle: function and clinical implications. J Vet Intern Med. 1994;8:73-78. 10.1111/j.1939-1676.1994.tb03201.x 8046679

[11] Gruskay JA . Inhibition of mitochondrial carbamyl phosphate synthetase in rat and human liver by ACYL CoA esters: a possible mechanism for hyperammonemia in the inherited organic acidemias. Yale Medicine Thesis Digital Library. 1981;2680. http://elischolar.library.yale.edu/ymtdl/2680

[12] Watson PJ, Herrtage ME. Medical management of congenital portosystemic shunts in 27 dogs--a retrospective study. J Small Anim Pract. 1998;39:62-68. 10.1111/j.1748-5827.1998.tb03595.x 9513885

[13] Tivers MS, Handel I, Gow AG, Lipscomb VJ, Jalan R, Mellanby RJ. Hyperammonemia and systemic inflammatory response syndrome predicts presence of hepatic encephalopathy in dogs with congenital portosystemic shunts. PloS One. 2014;9:e82303. 10.1371/journal.pone.0082303 PMC3879253 24392080

[14] Devriendt N, Serrano G, Paepe D, Vandenabeele S, Stock E, de Rooster H. Persistent hypercobalaminemia three months after successful gradual attenuation of extrahepatic shunts in dogs: a prospective cohort study. BMC Vet Res. 2022;18:18. 10.1186/s12917-021-03123-1 PMC8734341 34991571

[15] Arendt JF, Nexo E. Cobalamin related parameters and disease patterns in patients with increased serum cobalamin levels. PloS One. 2012;7:e45979. 10.1371/journal.pone.0045979 PMC3448722 23029349

[16] Da Riz F, Higgs P, Ruiz G. Diseases associated with hypercobalaminemia in dogs in United Kingdom: a retrospective study of 47 dogs. Can Vet J. 2021;62:611-616. PMC8118170 34219769

[17] Andrès E, Serraj K, Zhu J, Vermorken AJ. The pathophysiology of elevated vitamin B12 in clinical practice. Qjm. 2013;106:505-515. 10.1093/qjmed/hct051 23447660

[18] Herzlich B, Herbert V. Depletion of serum holotranscobalamin II. An early sign of negative vitamin B12 balance. Lab Invest. 1988;58:332-337. 3347009

[19] Devalia V, Hamilton MS, Molloy AM, the British Committee for Standards in Haematology. Guidelines for the diagnosis and treatment of cobalamin and folate disorders. Br J Haematol. 2014;166:496-513. 10.1111/bjh.12959 24942828

[20] Kather S, Reich F, Nexo E, et al. Serum transcobalamin analysis in dogs–method validation and association with serum cobalamin status in dogs with chronic enteropathy. J Vet Intern Med. 2022;36:30. 10.111/jvim.16559

[21] Kather S, Sielski L, Dengler F, Jirasek A, Heilmann RM. Prevalence and clinical relevance of hypercobalaminaemia in dogs and cats. The Veterinary Journal. 2020;265:105547. 10.1016/j.tvjl.2020.105547 33129556

[22] Kook PH, Drögemüller M, Leeb T, Howard J, Ruetten M. Degenerative liver disease in young beagles with hereditary cobalamin malabsorption because of a mutation in the cubilin gene. J Vet Intern Med. 2014;28:666-671. 10.1111/jvim.12295 PMC4858026 24467303

[23] Proksch AL, Schaefer S, Dreller V, et al. The effects of age and sex on reference intervals for cobalamin, homocysteine, and serum and urinary methylmalonic acid in healthy adult dogs. J Vet Intern Med. 2025;39:e17250. 10.1111/jvim.17250 PMC11647175 39676668

[24] Berghoff N, Suchodolski JS, Steiner JM. Association between serum cobalamin and methylmalonic acid concentrations in dogs. Vet J. 2012;191:306-311. 10.1016/j.tvjl.2011.03.005 21478039

[25] Lutz S, Sewell AC, Bigler B, Riond B, Reusch CE, Kook PH. Serum cobalamin, urine methylmalonic acid, and plasma total homocysteine concentrations in border collies and dogs of other breeds. Am J Vet Res. 2012;73:1194-1199. 10.2460/ajvr.73.8.1194 22849680

[26] Ullal TV, Marks SL, Huebner SN, Taylor SL, Shelley CD. Association of folate concentrations with clinical signs and laboratory markers of chronic enteropathy in dogs. J Vet Intern Med. 2023;37:455-464. 10.1111/jvim.16681 PMC10061186 36919188

[27] McLeish SA, Burt K, Papasouliotis K. Analytical quality assessment and method comparison of immunoassays for the measurement of serum cobalamin and folate in dogs and cats. J Vet Diagn Invest. 2019;31:164-174. 10.1177/1040638718824073 PMC6838819 30638139

[28] Baker H, Leevy CB, DeAngelis B, Frank O, Baker ER. Cobalamin (vitamin B12) and holotranscobalamin changes in plasma and liver tissue in alcoholics with liver disease. J Am Coll Nutr. 1998;17:235-238. 10.1080/07315724.1998.10718752 9627908

[29] Herrmann W, Obeid R, Schorr H, Geisel J. Functional vitamin B12 deficiency and determination of holotranscobalamin in populations at risk. Clin Chem Lab Med. 2003;41:1478-1488. 10.1515/CCLM.2003.227 14656029

[30] Liu Y, Gao Y, Liu Y, Zhang Y, Wang S, Yu B. Prevalence of functional cobalamin deficiency and relevant mortality risk in the general population: an unheeded phenotype distinct from cobalamin deficiency. J Am Nutr Assoc. 2025;44:181-189. 10.1080/27697061.2024.2412594 39405111

[31] Lindenbaum J, Savage DG, Stabler SP, Allen RH. Diagnosis of cobalamin deficiency: II. Relative sensitivities of serum cobalamin, methylmalonic acid, and total homocysteine concentrations. Am J Hematol. 1990;34:99-107. 10.1002/ajh.2830340205 2339684

[32] Briani C, Dalla Torre C, Citton V, et al. Cobalamin deficiency: clinical picture and radiological findings. Nutrients. 2013;5:4521-4539. 10.3390/nu5114521 PMC3847746 24248213

[33] Russell-Jones G . Paradoxical vitamin B12 deficiency: normal to elevated serum B12, with metabolic vitamin B12 deficiency. J Biol. 2022;11:001-004. 10.35248/2322-3308-11.3.003

[34] Demir N, Doğan M, Koç A, et al. Dermatological findings of vitamin B12 deficiency and resolving time of these symptoms. Cutan Ocul Toxicol. 2014;33:70-73. 10.3109/15569527.2013.861477 24303868

[35] Osifo BO, Ayoola A, Parmentier Y, Gerard P, Nicolas JP. Correlation between serum enzymes and serum unsaturated vitamin B12 binding proteins in primary liver carcinoma. Enzyme. 1988;39:161-166. 10.1159/000469112 2837386

[36] Couture Y, Keys D, Summers S. Comparison of hematologic variables between dogs with congenital intrahepatic and extrahepatic portosystemic shunts. J Vet Intern Med. 2024;38:1458-1464. 10.1111/jvim.17081 PMC11099786 38699882

[37] Ermens AA, Vlasveld LT, Lindemans J. Significance of elevated cobalamin (vitamin B12) levels in blood. Clin Biochem. 2003;36:585-590. 10.1016/j.clinbiochem.2003.08.004 14636871

[38] Alam A, Woo JS, Schmitz J, et al. Structural basis of transcobalamin recognition by human CD320 receptor. Nat Commun. 2016;7:12100. 10.1038/ncomms12100 PMC4947154 27411955

[39] Gick GG, Arora K, Sequeira JM, Nakayama Y, Lai SC, Quadros EV. Cellular uptake of vitamin B(12): role and fate of TCblR/CD320, the transcobalamin receptor. Exp Cell Res. 2020;396:112256. 10.1016/j.yexcr.2020.112256 32898552

[40] Soda R, Tavassoli M, Jacobsen DW. Receptor distribution and the endothelial uptake of transcobalamin II in liver cell suspensions. Blood. 1985;65:795-802. 10.1182/blood.V65.4.795.795 2983795

[41] Sobczak-Filipiak M, Męcik-Kronenberg T, Czopowicz M, et al. Lipogranulomas and pigment granulomas in livers of dogs with portosystemic shunt. Pol J Vet Sci. 2018;21:265-272. 10.24425/119047 30450864

[42] Salwei RM, O'Brien RT, Matheson JS. Use of contrast harmonic ultrasound for the diagnosis of congenital portosystemic shunts in three dogs. Vet Radiol Ultrasound. 2003;44:301-305. 10.1111/j.1740-8261.2003.tb00459.x 12816372

[43] Zwingenberger AL, Daniel L, Steffey MA, et al. Correlation between liver volume, portal vascular anatomy, and hepatic perfusion in dogs with congenital portosystemic shunt before and after placement of ameroid constrictors. Vet Surg. 2014;43:926-934. 10.1111/j.1532-950X.2014.12193.x 24724740

[44] Areekul S, Panatampon P, Doungbarn J. Vitamin B12 and vitamin B12 binding proteins in liver diseases. Southeast Asian J Trop Med Public Health. 1977;8:322-328. 607423

[45] Phillips RK, Steiner JM, Suchodolski JS, Lidbury JA. Urinary 15-F(2t)-Isoprostane concentrations in dogs with liver disease. Vet Sci. 2023;10. 10.3390/vetsci10020082 PMC9958836 36851386

[46] Kilpatrick S, Gow AG, Foale RD, et al. Plasma cytokine concentrations in dogs with a congenital portosystemic shunt. Vet J. 2014;200:197-199. 10.1016/j.tvjl.2014.01.007 24518641

[47] Devriendt N, Paepe D, Serrano G, et al. Plasma amino acid profiles in dogs with closed extrahepatic portosystemic shunts are only partially improved 3 months after successful gradual attenuation. J Vet Intern Med. 2021;35:1347-1354. 10.1111/jvim.16135 PMC8162604 33949730

[48] Bristow P, Tivers M, Packer R, et al. Long-term serum bile acid concentrations in 51 dogs after complete extrahepatic congenital portosystemic shunt ligation. J Small Anim Pract. 2017;58:454-460. 10.1111/jsap.12685 28660694

[49] Kather S, Grützner N, Kook PH, Dengler F, Heilmann RM. Review of cobalamin status and disorders of cobalamin metabolism in dogs. J Vet Intern Med. 2020;34:13-28. 10.1111/jvim.15638 PMC6979111 31758868

[50] Carmel R, Vasireddy H, Aurangzeb I, George K. High serum cobalamin levels in the clinical setting--clinical associations and holo-transcobalamin changes. Clin Lab Haematol. 2001;23:365-371. 10.1046/j.1365-2257.2001.00134.x 11843883

[51] Davis RE . Clinical chemistry of vitamin B12. Adv Clin Chem. 1985;24:163-216. 10.1016/s0065-2423(08)60273-5 3911750

[52] Chow BF, Okuda K. Transfer of vitamins from mother to FETUS. JAMA. 1960;172:422-426. 10.1001/jama.1960.03020050014005 13810038

[53] Abbas A, Snijders RJ, Nicolaides KH. Serum ferritin and cobalamin in growth retarded fetuses. Br J Obstet Gynaecol. 1994;101:215-219. 10.1111/j.1471-0528.1994.tb13112.x 8193095

[54] Bosle J, Goetz S, Raab A, et al. Cobalamin concentrations in Fetal liver show gender differences: a result from using a high-pressure liquid chromatography-inductively coupled plasma mass spectrometry as an Ultratrace cobalt speciation method. Anal Chem. 2016;88:12419-12426. 10.1021/acs.analchem.6b03730 28193074

[55] Konstantinidis AO, Adamama-Moraitou KK, Patsikas MN, Papazoglou LG. Congenital portosystemic shunts in dogs and cats: treatment, complications and prognosis. Vet Sci. 2023;10. 10.3390/vetsci10050346 PMC10223741 37235429

[56] Serrano G, Devriendt N, de Rooster H, Paepe D. Comparison of diet, lactulose, and metronidazole combinations in the control of pre-surgical clinical signs in dogs with congenital extrahepatic portosystemic shunts. J Vet Intern Med. 2022;36:1258-1266. 10.1111/jvim.16464 PMC9308425 35633289

[57] Stabler SP, Lindenbaum J, Allen RH. The use of homocysteine and other metabolites in the specific diagnosis of vitamin B-12 deficiency. J Nutr. 1996;126:1266s-1272s. 10.1093/jn/126.suppl_4.1266S 8642468

[58] Stabler SP, Marcell PD, Podell ER, Allen RH, Savage DG, Lindenbaum J. Elevation of total homocysteine in the serum of patients with cobalamin or folate deficiency detected by capillary gas chromatography-mass spectrometry. J Clin Invest. 1988;81:466-474. 10.1172/JCI113343 PMC329593 3339129

[59] Hortin GL, Seam N, Hoehn GT. Bound homocysteine, cysteine, and cysteinylglycine distribution between albumin and globulins. Clin Chem. 2006;52:2258-2264. 10.1373/clinchem.2006.074302 17068168

[60] McCarty MF, Barroso-Aranda J, Contreras F. The low-methionine content of vegan diets may make methionine restriction feasible as a life extension strategy. Med Hypotheses. 2009;72:125-128. 10.1016/j.mehy.2008.07.044 18789600

[61] Center SA . Nutritional support for dogs and cats with hepatobiliary disease. J Nutr. 1998;128:S2733-S2746. 10.1093/jn/128.12.2733S 9868254

[62] Gibson EA, Culp WTN, Hardy BT, et al. Subclinical gastrointestinal mucosal lesions occur in dogs with intrahepatic portosystemic shunts independent of percutaneous transvenous coil embolization. J Am Vet Med Assoc. 2025;263:1011-1018. 10.2460/javma.24.11.0707 40010298

[63] Weisse C, Berent AC, Todd K, Solomon JA, Cope C. Endovascular evaluation and treatment of intrahepatic portosystemic shunts in dogs: 100 cases (2001–2011). J Am Vet Med Assoc. 2014;244:78-94. 10.2460/javma.244.1.78 24344856

[64] Frymus J, Trębacz P, Galanty M. Gastrointestinal ulceration in dogs with portosystemic shunt. *Med Weter*. 2023;79:288-292. 10.21521/mw.6786

[65] O'Kell AL, Gallagher AE, Cooke KL. Gastroduodenal ulceration in dogs with liver disease. J Vet Intern Med. 2022;36:986-992. 10.1111/jvim.16413 PMC9151467 35312117

[66] Evenepoel P, Claus D, Geypens B, et al. Evidence for impaired assimilation and increased colonic fermentation of protein, related to gastric acid suppression therapy. Aliment Pharmacol Ther. 1998;12:1011-1019. 10.1046/j.1365-2036.1998.00377.x 9798807

[67] Mosharov E, Cranford MR, Banerjee R. The quantitatively important relationship between homocysteine metabolism and glutathione synthesis by the transsulfuration pathway and its regulation by redox changes. Biochemistry. 2000;39:13005-13011. 10.1021/bi001088w 11041866

[68] Vitvitsky V, Mosharov E, Tritt M, Ataullakhanov F, Banerjee R. Redox regulation of homocysteine-dependent glutathione synthesis. Redox Rep. 2003;8:57-63. 10.1179/135100003125001260 12631446

[69] Lord RS, Kara Fitzgerald N. Significance of Low Plasma Homocysteine. USA: Metametrix Laboratory Department of Science and Education. URL: http://www.peirsoncenter.com/uploads/6/0/5/5/6055321/significance-of-low-plasma-homocysteine.pdf; 2006.

[70] Huemer M, Vonblon K, Födinger M, et al. Total homocysteine, folate, and cobalamin, and their relation to genetic polymorphisms, lifestyle and body mass index in healthy children and adolescents. Pediatr Res. 2006;60:764-769. 10.1203/01.pdr.0000246099.39469.18 17065574

[71] González-Gross M, Benser J, Breidenassel C, et al. Gender and age influence blood folate, vitamin B12, vitamin B6, and homocysteine levels in European adolescents: the Helena study. Nutr Res. 2012;32:817-826. 10.1016/j.nutres.2012.09.016 23176792

[72] De Laet C, Wautrecht JC, Brasseur D, et al. Plasma homocysteine concentration in a Belgian school-age population. Am J Clin Nutr. 1999;69:968-972. 10.1093/ajcn/69.5.968 10232638

[73] Rauh M, Verwied S, Knerr I, Dörr HG, Sönnichsen A, Koletzko B. Homocysteine concentrations in a German cohort of 500 individuals: reference ranges and determinants of plasma levels in healthy children and their parents. Amino Acids. 2001;20:409-418. 10.1007/s007260170037 11452984

[74] Brattström L, Lindgren A, Israelsson B, Andersson A, Hultberg B. Homocysteine and cysteine: determinants of plasma levels in middle-aged and elderly subjects. J Intern Med. 1994;236:633-641. 10.1111/j.1365-2796.1994.tb00856.x 7989898

[75] Shohag MJ, Wei Y, Yu N, et al. Folate content and composition of vegetables commonly consumed in China. J Food Sci. 2012;77:H239-H245. 10.1111/j.1750-3841.2012.02939.x 23127121

[76] Wang A, Yeung LF, Ríos Burrows N, et al. Reduced kidney function is associated with increasing red blood cell folate concentration and changes in folate form distributions (NHANES 2011–2018). Nutrients. 2022;14:1054. 10.3390/nu14051054 PMC8912286 35268029

[77] Smulders Y, Smith D, Kok R, et al. Cellular folate vitamer distribution during and after correction of vitamin B12 deficiency: a case for the methylfolate trap. Br J Haematol. 2006;132:623-629. 10.1111/j.1365-2141.2005.05913.x 16445837

[78] Minović I, Dikkeschei LD, Vos MJ, Kootstra-Ros K-R. Interpretation of folate results in hemolytic plasma samples: a practical approach. Ann Lab Med. 2021;41:485-488. 10.3343/alm.2021.41.5.485 PMC8041598 33824237

[79] Batt R, Morgan J. Role of serum folate and vitamin B12 concentrations in the differentiation of small intestinal abnormalities in the dog. Res Vet Sci. 1982;32:17-22. 10.1016/S0034-5288(18)32430-5 7089378

[80] Rutgers HC, Batt RM, Elwood CM, Lamport A. Small intestinal bacterial overgrowth in dogs with chronic intestinal disease. J Am Vet Med Assoc. 1995;206:187-193. 10.2460/javma.1995.206.02.187 7751219

[81] Squire N, Lux C, Tolbert K, Lidbury J, Sun X, Suchodolski JS. Characterization of the Fecal microbiome in dogs receiving medical Management for Congenital Portosystemic Shunts. Front Vet Sci. 2022;9:897760. 10.3389/fvets.2022.897760 PMC9366551 35968011

[82] Allen RH, Stabler SP, Savage DG, Lindenbaum J. Diagnosis of cobalamin deficiency I: usefulness of serum methylmalonic acid and total homocysteine concentrations. Am J Hematol. 1990;34:90-98. 10.1002/ajh.2830340204 2339683

[83] Supakul S, Chabrun F, Genebrier S, et al. Diagnostic performances of urinary Methylmalonic acid/creatinine ratio in vitamin B12 deficiency. J Clin Med. 2020;9:S1-11. 10.3390/jcm9082335 PMC7466029 32707915

[84] Ganji V, Kafai MR. Population reference values for serum Methylmalonic acid concentrations and its relationship with age, sex, race-ethnicity, supplement use, kidney function and serum vitamin B12 in the post-folic acid fortification period. Nutrients. 2018;10:1-13. 10.3390/nu10010074 PMC5793302 29329201

[85] Zhang Z, Lv L, Guan S, et al. Association between serum methylmalonic acid and chronic kidney disease in adults: a cross-sectional study from NHANES 2013-2014. Front Endocrinol (Lausanne). 2024;15:1434299. 10.3389/fendo.2024.1434299 PMC11324440 39149121

[86] Kook PH, Hersberger M. Daily oral cyanocobalamin supplementation in beagles with hereditary cobalamin malabsorption (Imerslund-Gräsbeck syndrome) maintains normal clinical and cellular cobalamin status. J Vet Intern Med. 2019;33:751-757. 10.1111/jvim.15380 PMC6430909 30554416

[87] Jimenez L, Stamm DA, Depaula B, Duggan CP. Is serum methylmalonic acid a reliable biomarker of vitamin B12 status in children with short bowel syndrome: a case series. J Pediatr. 2018;192:259-261. 10.1016/j.jpeds.2017.09.024 PMC6029886 29129351

[88] Sentongo TA, Azzam R, Charrow J. Vitamin B12 status, methylmalonic acidemia, and bacterial overgrowth in short bowel syndrome. J Pediatr Gastroenterol Nutr. 2009;48:495-497. 10.1097/MPG.0b013e31817f9e5b 19322060

[89] Burlina A, Tims S, van Spronsen F, et al. The potential role of gut microbiota and its modulators in the management of propionic and methylmalonic acidemia. Expert Opin Orphan Drugs. 2018;6:683-692. 10.1080/21678707.2018.1536540

[90] Rasmussen K, Moelby L, Jensen MK. Studies on methylmalonic acid in humans. II. Relationship between concentrations in serum and urinary excretion, and the correlation between serum cobalamin and accumulation of methylmalonic acid. Clin Chem. 1989;35:2277-2280. 10.1093/clinchem/35.12.2277 2591043

[91] Flatley JE, Garner CM, Al-Turki M, et al. Determinants of urinary methylmalonic acid concentration in an elderly population in the United Kingdom. Am J Clin Nutr. 2012;95:686-693. 10.3945/ajcn.111.024133 22301932

[92] Deppe TA, Center SA, Simpson KW, et al. Glomerular filtration rate and renal volume in dogs with congenital portosystemic vascular anomalies before and after surgical ligation. J Vet Intern Med. 1999;13:465-471. 10.1111/j.1939-1676.1999.tb01464.x 10499731

[93] Holden HM, Thoden JB, Raushel FM. Carbamoyl phosphate synthetase: an amazing biochemical odyssey from substrate to product. Cell Mol Life Sci. 1999;56:507-522. 10.1007/s000180050448 PMC11147029 11212301

[94] Devriendt N, Serrano G, Stock E, Paepe D, de Rooster H. Diagnostic value of liver function tests and ultrasonography in dogs with suspected congenital portosystemic shunts. Vet Rec. 2022;190:e1381. 10.1002/vetr.1381 35102558

[95] Kim SE, Giglio RF, Reese DJ, Reese SL, Bacon NJ, Ellison GW. Comparison of computed tomographic angiography and ultrasonography for the detection and characterization of portosystemic shunts in dogs. Vet Radiol Ultrasound. 2013;54:569-574. 10.1111/vru.12059 23763372

[96] Fulton RM . Focused—basic ultrasound principles and artifacts. In: Lisciandro GR, ed. Focused ultrasound techniques for the small animal practitioner. Wiley; 2014:1-16.

[97] d'Anjou MA, Penninck D, Cornejo L, Pibarot P. Ultrasonographic diagnosis of portosystemic shunting in dogs and cats. Vet Radiol Ultrasound. 2004;45:424-437. 10.1111/j.1740-8261.2004.04076.x 15487568

[98] Devriendt N, Paepe D, Serrano G, de Rooster H. Evaluation of different blood tests in dogs with extrahepatic portosystemic shunts to assess shunt closure after surgical treatment. Vet Surg. 2022;51:1142-1152. 10.1111/vsu.13840 35729849

[99] Elango R . Methionine nutrition and metabolism: insights from animal studies to inform human nutrition. J Nutr. 2020;150:2518s-2523s. 10.1093/jn/nxaa155 33000159

